# Global monitoring of methane point sources using deep learning on hyperspectral radiance measurements from EMIT

**DOI:** 10.1073/pnas.2612145123

**Published:** 2026-09-01

**Authors:** Vishal V. Batchu, Michelangelo Conserva, Alex Wilson, Anna M. Michalak, Varun Gulshan, Philip G. Brodrick, Andrew K. Thorpe, Christopher V. Arsdale

**Affiliations:** ^a^ Google Research, Google, Mountain View, CA 94043, USA; ^b^ Division of Biosphere Sciences and Engineering, Carnegie Institution for Science, Washington, DC 20015; ^c^ Jet Propulsion Laboratory, California Institute of Technology, Pasadena, CA 91109

**Keywords:** vision transformer, Earth Surface Mineral Dust Source Investigation, imaging spectroscopy, methane plume mapping, greenhouse gas mitigation

## Abstract

Anthropogenic methane (CH_4_) point sources are critical drivers of near-term climate forcing, safety hazards, and system-inefficiencies. Space-based imaging spectroscopy is an emerging tool for identifying emissions globally, but existing approaches largely rely on manual plume identification. Here, we present the Methane Analysis and Plume Localization with Earth Surface Mineral Dust Source Investigation (MAPL-EMIT) model, an end-to-end vision transformer framework that leverages the complete radiance spectrum from the EMIT instrument to jointly retrieve methane enhancements across all pixels within a scene. This approach brings together spectral information with spatial context to significantly lower detection limits. MAPL-EMIT simultaneously supports enhancement quantification, plume delineation, and source localization, even for overlapping plumes. The model was trained on 3.6 million physics-based synthetic plumes injected into global EMIT radiance data. Synthetic evaluation confirms the model’s ability to identify plumes with high recall and precision and to capture weaker plumes relative to existing matched-filter approaches. On real-world benchmarks, MAPL-EMIT captures 84% of known hand-annotated NASA EMIT-L2B plume complexes across a test set of 1,084 EMIT granules, while capturing roughly 1.5 times as many plausible plumes compared to human analysts. Further verification against coincident airborne data, top-emitting landfills, and controlled release experiments confirms the model’s ability to identify previously uncaptured sources. By incorporating model-generated metrics such as spectral fit scores and estimated noise levels, the framework can limit false-positives. Overall, MAPL-EMIT enables high-throughput implementation on the full EMIT data catalog, shifting methane monitoring to a rapid, scalable paradigm for global plume mapping at the facility scale.

## Significance

Methane point sources drive near-term climate forcing, yet tracking them globally remains challenging. We present a deep-learning framework that advances emission monitoring by directly analyzing satellite-based spectroscopic data and demonstrate its application to data from the Earth Surface Mineral Dust Source Investigation (EMIT) instrument. This approach provides the spatial context required to map gas concentrations across landscapes, significantly lowering detection limits and automating plume delineation and source localization, even for overlapping methane plumes from multiple sources. We validate the model against synthetic and real-world benchmarks, and demonstrate that it captures known emitters while identifying numerous previously undetected plumes. The automated end-to-end modeling pipeline enables application to the full EMIT archive, providing a compelling foundation for climate mitigation and facility-level accountability.

Atmospheric methane (CH_4_) is a potent climate-forcing agent and the second largest contributor to anthropogenic greenhouse gas warming. With a 20-y global warming potential 81 to 86 times that of CO_2_, CH_4_ has driven approximately 25% of human-induced warming since the start of the industrial era. Given its high radiative efficiency and a relatively short 9-y atmospheric lifetime (1), reducing CH_4_ emissions offers a critical “fast-action” pathway to mitigating global temperature rise. This urgency is reflected in the Global Methane Pledge (2), under which over 125 countries have committed to a 30% reduction in emissions by 2030.

Achieving these targets requires reliable monitoring of anthropogenic sources, which account for 60% of global CH_4_ output. Within this 60%, emissions are concentrated within the waste (19%), agriculture (44%), and energy sectors (37%). The energy sector emissions consist of oil (33%), gas (25%), coal (28%), and bioenergy (14%) (3). Critically, much of the energy sector’s footprint originates from localized point sources, making them primary targets for cost-effective mitigation.

Current space-based remote sensing of methane emissions faces a fundamental trade-off: instruments like the TROPOspheric Monitoring Instrument (TROPOMI) provide daily global coverage but at a coarse spatial resolution of 5.5 × 3.5 km^2^ (4) unsuitable for isolating individual sources. Conversely, targeted commercial imagers like GHGSat offer a high spatial resolution of 25 × 25 m^2^ but very limited spatial coverage and restricted data access (5). While multispectral sensors (e.g., Sentinel-2, Landsat) bridge this gap (6), they are limited to capturing very large emissions due to their coarse spectral resolution.

This study contributes to the growing literature that leverages observations from the Earth Surface Mineral Dust Source Investigation (EMIT) (7) to address this gap. Although originally designed for mineral mapping, EMIT’s pushbroom hyperspectral imaging spectrometer balances these competing needs by providing high spectral and spatial resolution with substantial spatial coverage. Deployed on the International Space Station (ISS), EMIT captures 285 spectral bands (381 to 2,493 nm) in the visible through Short-Wave Infrared (SWIR) with a 60 m spatial resolution and an 80 km swath. This configuration provides the high Signal-to-Noise Ratio (SNR) and broad surface coverage necessary to characterize fine-scale methane absorption features across large regions. Existing approaches primarily rely on the Adaptive Matched Filter (AMF) (8–10) for characterizing methane enhancements, followed by either expert-guided (e.g., refs. 7 and 11) or machine-learning-based (e.g., ref. 12) plume segmentation. False positives caused by surface clutter, nonhomogeneous terrain, and retrieval artifacts require careful examination, which presents challenges when scaling to global monitoring. Deep learning models built on the underlying radiance data but which also include a spatial field via Convolutional Neural Networks (CNNs) or Vision Transformers, on the other hand, provide a potential scalable alternative (6, 13–16). The core of the value added by these methods is the joint utilization of spectral characteristics and spatial patterning of those characteristics within the scene, which has the potential to suppress background noise and lower detection limits. The use of these methods is currently bottlenecked by a scarcity of diverse, high-quality annotated real-world data, however. While some studies have attempted to leverage synthetic plumes superimposed on real backgrounds (6, 17), these efforts have largely been limited to multispectral sensors, small-scale datasets, or simplified radiative transfer models that struggle to generalize to complex operational environments.

Here we introduce the Methane Analysis and Plume Localization with EMIT (MAPL-EMIT) model, which directly utilizes the complete EMIT radiance spectrum in a vision transformer, providing spatial context to identify methane plumes across scenes. The model is trained on 3.6 million synthetic methane plumes superimposed on real EMIT backgrounds. The approach is used to automate enhancement quantification, plume segmentation, and source localization, including for multiple overlapping plumes, unlocking the capability to rapidly investigate the full EMIT data record. We assess the model’s ability to lower the detection limit for methane point sources. We further evaluate the model against a diverse set of real-world benchmarks, including plumes identified in the scientist-annotated NASA EMIT L2B product (18), an inventory of top-emitting landfills globally, coincident high-resolution airborne observations, and controlled release intercomparison studies.

## Results

### Evaluation on Synthetic Plumes.

Evaluation against synthetic observations confirms the ability of MAPL-EMIT to detect and quantify methane enhancements, delineate plumes, and identify source location with high recall and precision.

**Fig. 1.**
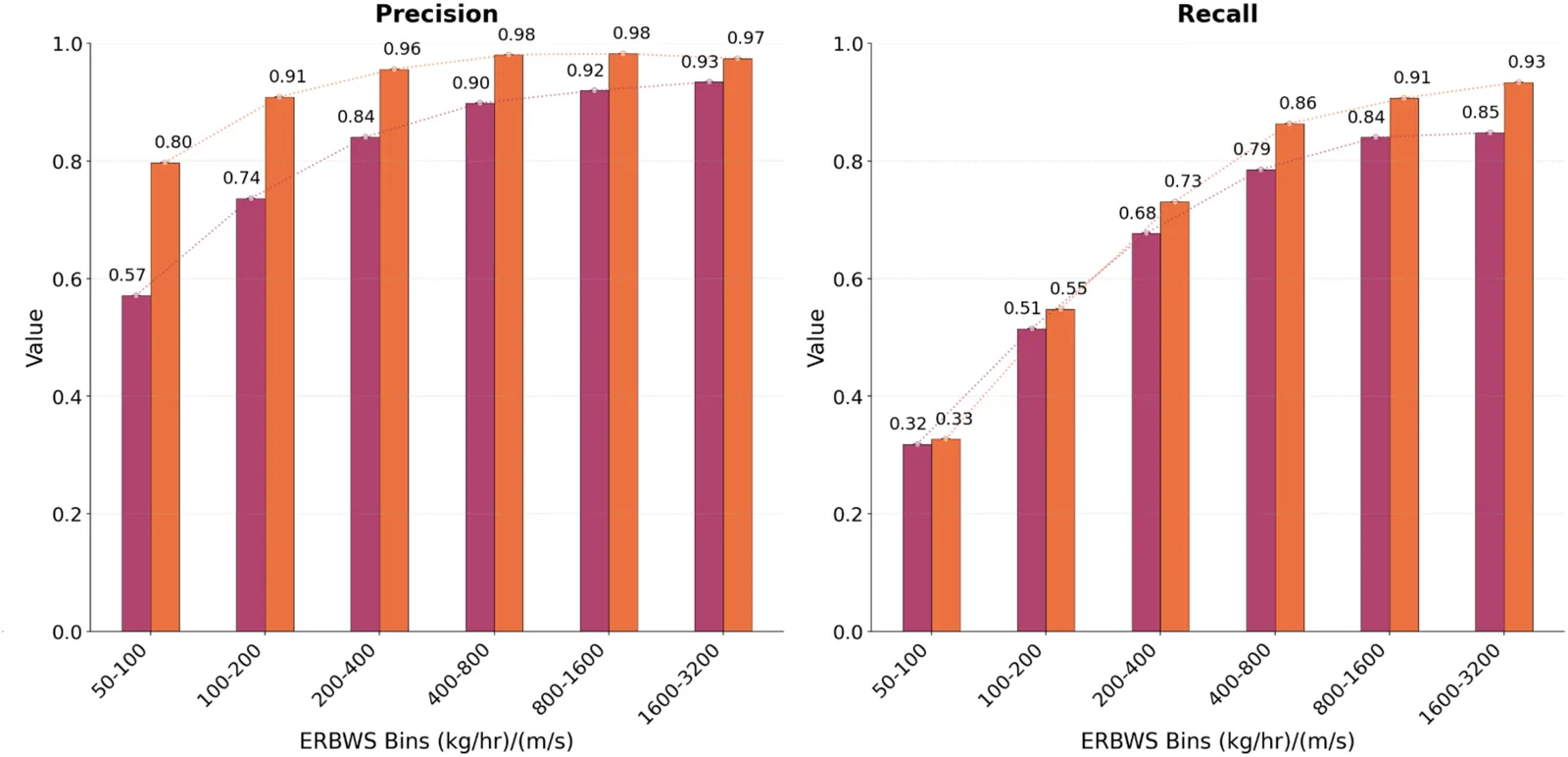
MAPL-EMIT exhibits high precision and recall across plumes with a wide range of emission rates on the synthetic plume test split. Precision (*Left* panel) and recall (*Right* panel) are binned for different plume intensity levels, defined as emission rate (kg/h) divided by 10 m elevation wind speed (m/s) (ERBWS), for scenes with single plumes (orange) as well as for multiple overlapping plumes (in the same EMIT tile) (magenta). The numbers above the bars indicate the precision and recall for each ERBWS bin.

For detection of individual plumes, the model precision increases from 0.80 for the weakest plumes [50 to 100 (kg/h)/(m/s) which is equivalent to 125 to 250 kg/h at typical wind speeds of ~2.5 m/s] to 0.97 for the most intense plumes [1,600 to 3,200 (kg/h)/(m/s)], indicating a decreasing rate of false positives as the plume intensity increases (Fig. 1). The model recall ranges from 0.33 for the weakest plumes to 0.93 for the most intense ones, indicating an increasing ability to detect plumes at higher intensities, as expected.

The model also demonstrated high overall enhancement quantification accuracy on the synthetic test set. The normalized root mean squared error (RMSE) ranged from 6% for the highest intensity pixels (>2,500 ppm-m) to 26% for pixels with enhancements in the range of 250 to 500 ppm-m, and greater for weaker enhancements (*SI Appendix*, Fig. S1). The symmetric mean absolute percentage error (SMAPE) followed a similar pattern (*SI Appendix*, Fig. S1). Plume-level integrated enhancement metrics (*SI Appendix*, Fig. S2) show that pixel-level errors often cancel out, leading to lower plume-level errors ranging from 8% for the highest emission plumes [1,600 to 3,200 (kg/h)/(m/s)] to 24% for the weakest plumes [50 to 100 (kg/h)/(m/s)], confirming strong quantification performance.

The model’s ability to infer the source location was also strong, with a mean distance of 101 m (1.68 pixels) between the true and estimated source location when averaged across all the different examined plume intensities.

The model is able to successfully identify and segment multiple overlapping plumes while estimating multiple source locations, with only modest impacts on plume detection (Fig. 1) and enhancement quantification (*SI Appendix*, Fig. S1) for all but the weakest plumes. There was no impact on the ability of the model to identify the source location, with a mean distance between the true and estimated source location of 103 m or 1.72 pixels when averaged across all examined plume intensities. An example of this is presented in (*SI Appendix*, Fig. S3). This is especially beneficial in industrial regions, where proximal emissions often cause plumes to coalesce downwind of emissions sources. Such overlapping plumes are not well captured by existing approaches.

The synthetic plume results suggest that MAPL-EMIT is able to capture weaker plumes relative to existing approaches, thereby presenting the potential to lower the detection limit of methane point sources. In the following sections we assess the degree to which these results generalize to real-world plumes.

### Performance on Real-World Benchmarks.

We evaluate MAPL-EMIT against a wide range of data products to explore the model’s ability to automatically detect and delineate real methane plumes and to localize sources. Because no reference set is complete, we rely on the combination of these comparisons to provide confidence in the model’s performance. The comparisons include a comparison to EMIT L2B hand-annotated methane plume complexes (18), an examination of EMIT observations near the world’s 25 top-emitting landfills (19), an evaluation against coincident airborne observations from AVIRIS-3 (13) and GAO (20), and validation against coincident controlled releases (21).

#### NASA EMIT L2B plume complexes.

To validate detection capabilities against established benchmarks, we compare MAPL-EMIT’s plume detections and plume boundaries against the EMIT L2B estimated methane plume complexes v002 (18), (henceforth referred to as EMIT L2B plumes) across identical data granules. Model inference was run on 1,084 granules, which contained 1,580 EMIT L2B plumes. Because the EMIT L2B plumes have gone through multiple rounds of expert evaluation, the assumption is that these plumes contain relatively few false positives. Conversely, it is generally understood that many plumes are likely to be missed by this product due to background noise in the matched filter enhancements and due to a conscious decision to limit plumes to those with the highest confidence.

**Fig. 2.**
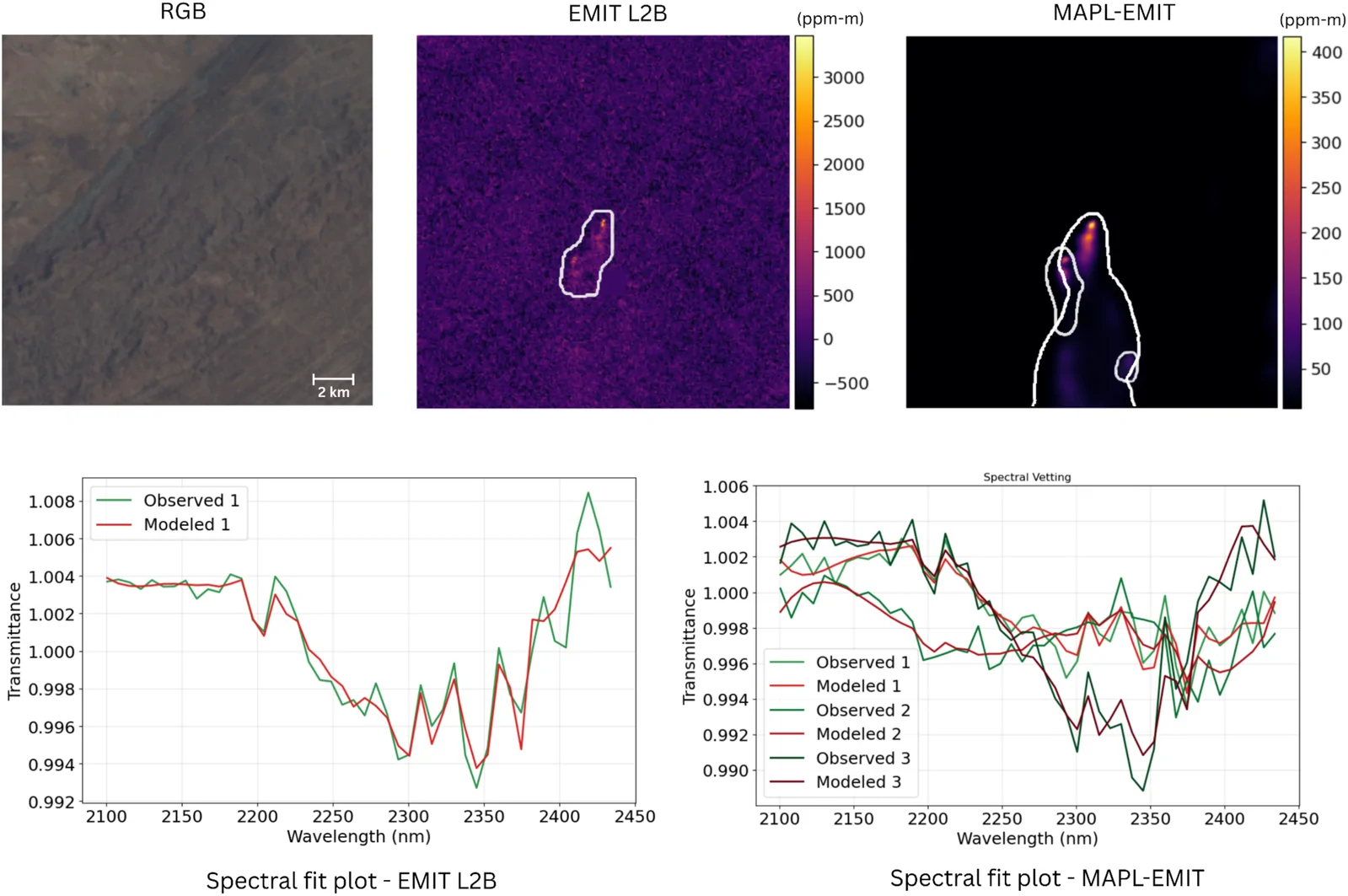
MAPL-EMIT detection and disambiguation of overlapping methane plumes. (*Top* row) RGB (*Left*), NASA L2B enhancements with plume masks (*Middle*), and MAPL-EMIT enhancements with plume delineation (*Right*). (*Bottom* row) Spectral fits provide physical verification. Observed transmittance ratios align with modeled methane absorption manifolds, supporting plume authenticity. While the first MAPL-EMIT plume shows a robust spectral fit, the second appears less definitive, likely due to its weaker intensity.

**Fig. 3.**
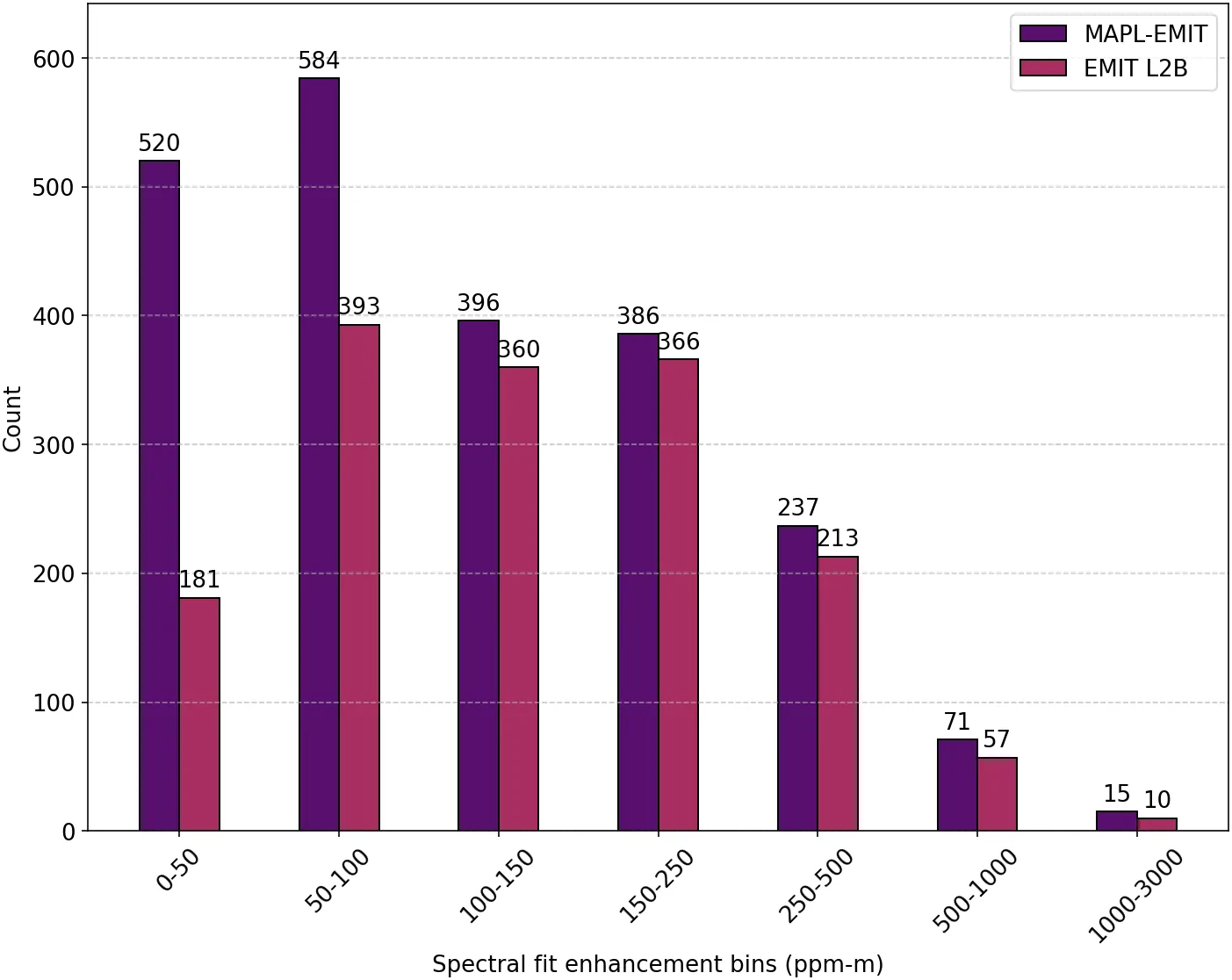
MAPL-EMIT identifies more potential plumes than the official EMIT L2B plume complexes. Plumes are binned by spectral fit enhancement (*Methods*). MAPL-EMIT (purple) consistently identifies more plumes across all intensities, specifically more than 2× the number of detections for the 0 to 50 ppm-m bin. The numbers above the bars indicate the number of identified plumes in each enhancement bin.

In order to focus on the higher confidence plumes identified by the MAPL-EMIT model, we filtered the model-identified plumes to those on-land (dropping any detections over water) and to those that appear in >13 of 16 occurrences when processing overlapping tiles (i.e., strided inference), where the clustering threshold of 13 corresponds to the number of detections of a plume by the model over 16 strided inferences (as detailed in the *SI Appendix*) (i.e., detection in >81.25% of strided inferences). MAPL-EMIT initially identified 3,672 plumes across the 1,084 granules used in the comparison with the EMIT L2B plumes, and this number was reduced to 2,209 after this filter was applied. An example tile from a granule is shown in Fig. 2. Spectral fits confirm the presence of true plumes. Enhancement values between the methods disagree here, though there are many plausible explanations for this for a single plume, ranging from matched-filter sensitivities (22) to enhancement misestimation. The presence of high noise pixels in matched filter enhancements, including substantial negative values and anomalously high positive values, particularly near the plume source, dictate the visual scale. We do however see that across plumes overall, MAPL-EMIT enhancements align better with spectral fit enhancement estimates (*Discussion*). The number of plumes for both models is broken out by the spectral fit enhancement in Fig. 3. While MAPL-EMIT identifies more plumes across all intensities, the increase in the number of detections is significantly higher in the lower 0-100 ppm-m bins.

An evaluation of individual plumes shows that the final set of MAPL-EMIT plumes captures 84% of the EMIT L2B plume complexes. To prevent small boundary disagreements compromising statistics, MAPL-EMIT plumes included a 0.01° (~1.1 km, ~18 pixel) buffer, and a capture was considered successful if there was any plume intersection; results were very similar however with no buffer. This confirms the ability of the MAPL-EMIT model to automatically identify and delineate the high-intensity emissions detected by a matched-filter algorithm and expert-guided hand-annotation.

Conversely, only 67% of the MAPL-EMIT plumes were captured in the EMIT L2B plume collection (using the same buffering strategy as above), indicating that MAPL-EMIT identified ~1.5x as many plumes as human analysts did. This is likely due to a combination of three factors. First, MAPL-EMIT appears to be able to identify weak plumes that are difficult to differentiate from background noise in matched-filter-based approaches, and which are therefore not present in the EMIT collection (*SI Appendix*, Fig. S4). This presents the possibility of identifying many more real plumes relative to existing approaches. The second factor is that the EMIT L2B product may even exclude high-intensity plumes due to stringent confidence thresholds in the review process or potential human omission during assessment (*SI Appendix*, Fig. S5). The third factor is that some of the MAPL-EMIT plumes are likely to be false positives, but the exact rate of false positives is challenging to assess given the lack of an absolute ground-truth. We examine this third factor next.

#### False positive rate assessment in nonemission granules.

Quantifying false-positive (FP) rates in real-world satellite observations is challenging due to the absence of globally verified “zero-emission” ground-truth datasets. To address this, we evaluated MAPL-EMIT’s performance on 20,000 granules where NASA JPL expects minimal plumes to be present. Per the EMIT L2B Greenhouse Gas ATBD (23), care is given to avoid false positives in plume delineation, and, as a result, some plumes are inevitably not included within this catalog. Also, human analysts may sometimes fail to identify some plumes. Therefore, while a reasonable indicator, this is not an exhaustive metric.

The model detected 1,513 distinct plume instances across these 20,000 granules post filtering the model-identified plumes to those on-land and with an inference cluster size >13 per plume. To differentiate plausible low-intensity emissions from likely false positives (such as retrieval artifacts or surface clutter), we followed the approach of Xiang et al. (24) (*Methods: Spectral Fit Comparison*) to conduct spectral fit evaluation on all detections. This approach allows us to assess plumes and identify potential false positives. In doing so, we found that 355 of these detections are likely to represent genuine methane plumes.

The remaining 1,158 unconfirmed detections, or approximately 0.06 plumes per 120 × 120 km^2^ granule, provide a conservative estimate of the model’s false-positive rate in complex real-world terrains. A majority of these plumes are on hilly terrain–both low and high albedo–or dense forests. By framing these unconfirmed detections as a conservative upper limit of the false positive rate, we demonstrate that the model maintains high precision while substantially expanding the envelope of detectable methane emissions.

#### High-emitting landfills.

Landfills present a challenging retrieval environment for methane detection due to high surface heterogeneity and variable albedo. Landfills also often include multiple emission sources and/or spatially diffuse emissions, making methane enhancement detection and plume delineation difficult for existing algorithms. Zhang et al. (19) had previously reported that the EMIT L2B methane complex product was able to detect potential plumes at 17 of the 25 top-emitting landfills globally.

Applying MAPL-EMIT at the locations of the 25 landfills demonstrated strong performance under these complex conditions. The model was able to identify potential plumes at 24 of the 25 landfills (examples in *SI Appendix*, Fig. S6). Importantly, the model had no information about underlying surface infrastructure or predominant wind direction and the correct identification of methane enhancements over these landfills is solely based on EMIT observations. In addition, MAPL-EMIT was often able to identify plumes for multiple EMIT overpasses, with the plume directions aligning with predominant wind directions at the time of the overpasses. An example of a site in Amman, Jordan, is shown in *SI Appendix*, Fig. S7. This location is a known persistent point source (25). MAPL-EMIT robustly captures persistent emission events across the full EMIT time series while avoiding false-positive detections in the surrounding area. Furthermore, the model resolves fine-grained plume morphology, capturing realistic, turbulent gas dispersion patterns. Here again, the inference was based solely on EMIT radiance information and wind information was not available to the model.

The application to landfills points to several additional strengths of MAPL-EMIT. First, as shown in *SI Appendix*, Fig. S6, the model demonstrates robust detection under partial cloud cover, a regime where traditional matched-filter methods often produce artifacts requiring the outputs to be masked out. Second, the fact that MAPL-EMIT is able to identify plumes, which originate at the landfill and are oriented along the predominant wind direction, for all but one of the top-emitting landfills, a substantially higher fraction than in the case of the EMIT L2B plumes (*SI Appendix*, Fig. S8), further supports the suggestions that MAPL-EMIT is able to successfully automatically detect weaker enhancements and delineate weaker plumes relative to existing approaches.

#### Airborne observations.

We leveraged airborne data with concurrent EMIT overpasses to further evaluate performance on real-world plumes. AVIRIS-3 served as the primary source for verification against airborne observations due to the availability of coincident overpasses (13). We analyzed an EMIT granule overlapping with several AVIRIS-3 acquisitions (26), which identified five plumes. With a native spatial resolution of 0.3 m for this set of aerial collects, AVIRIS-3 was a robust verification source for lower-intensity emissions often obscured in spatially coarser satellite data.

**Fig. 4.**
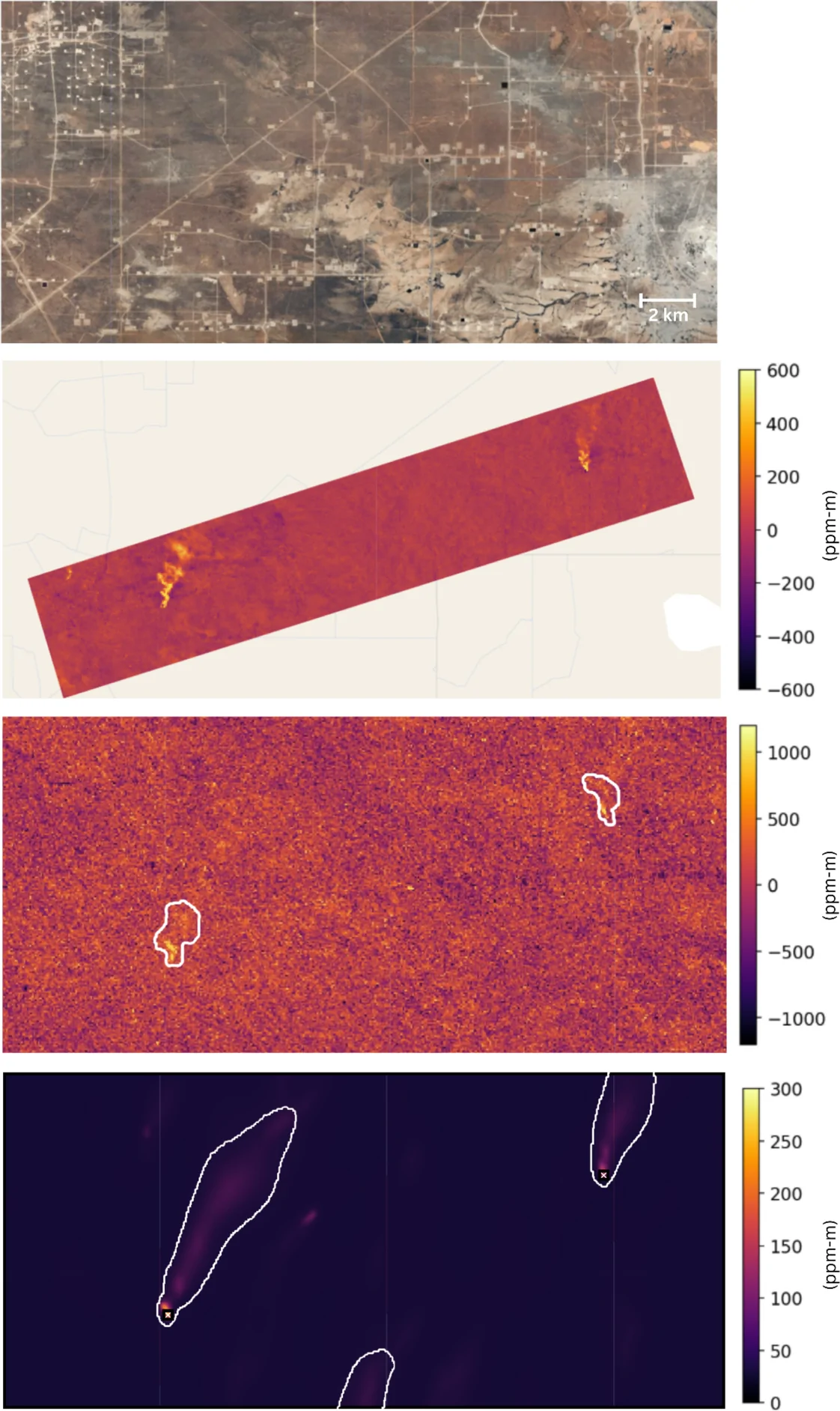
Enhanced signal-to-noise ratio and verification of MAPL-EMIT plumes against high-resolution aerial enhancements from AVIRIS-3. The panels display (from *Top* to *Bottom*): RGB, coincident AVIRIS-3 airborne observations resampled to 60 m resolution, NASA L2B enhancement with identified plume complexes on *Top*, where plumes are hard to distinguish from background noise, and MAPL-EMIT enhancements and plumes, which successfully suppress surface artifacts and accurately predict plume morphologies aligned with AVIRIS-3 observations.

We restricted the analysis to acquisitions collected within 30 min of the EMIT overpass. After resampling the aerial data to EMIT’s 60 m resolution, only three of the five original plumes remained discernible in the matched filter enhancements based on AVIRIS-3 data; the others became indistinguishable at the coarser scale (Fig. 4, second panel). MAPL-EMIT successfully detected two of the three resolvable plumes without generating false positives (Fig. 4, fourth panel) with an SNR of 8.60, higher than the SNR of resampled AVIRIS-3 matched filter which stood at 1.21. While the NASA L2B plumes also captured these plumes, a lower SNR of 0.40 in the standard matched-filters rendered them harder to isolate from background noise (Fig. 4, third panel). SNR is computed as the plume enhancement mean minus background enhancement mean divided by the SD of the background enhancement across the entire AVIRIS overpass (depicted in Fig. 4, second panel). While EMIT’s low-resolution constrains morphological precision, MAPL-EMIT better replicates AVIRIS-3 plume geometry and orientation than EMIT L2B. This is most evident in the second plume, where L2B deviates significantly at the tail.

A comparison against airborne observations from the Global Airborne Observatory (GAO) (20) using the same 30-min temporal constraint for coincident observations yielded similarly strong results (*SI Appendix*, Fig. S9).

#### Controlled releases.

We analyzed controlled methane release experiments with coincident EMIT overpasses (27), which included five releases from Phase 2 and two releases from Phase 4 of the experiments. Phases 1 and 3 lacked unmasked EMIT overpasses.

MAPL-EMIT successfully detected five of the seven plumes (*SI Appendix*, Fig. S10), with a sixth plume showing up in the enhancements but not being identified as a plume due to not meeting the required threshold for plume instance probabilities (*Methods*). Additionally, MAPL-EMIT did not produce any false positives at the location of release across any other EMIT overpasses of the locations. This detection rate confirms the model’s ability to capture short-duration medium-intensity releases [~300 to 1,000 (kg/h)/(m/s)]. Furthermore, spectral fit metrics across the detected plumes indicated strong agreement for most plumes (*SI Appendix*, Fig. S10, third column). Enhancements derived from these spectral fits (fit_enh) are also similar to enhancements predicted by the model (obs_enh) (*SI Appendix*, Fig. S10, fourth column).

## Discussion

### Challenges of Methane Emissions Detection.

Satellite methane detection faces four primary challenges. First, the lack of dedicated plume-detection instruments providing fully open radiance data. Most sensors that are presently used are not optimized for methane detection, being coarser either spatially (e.g., TROPOMI) or spectrally (e.g., EMIT, PRISMA, EnMAP) than would be ideal. Second, a severe scarcity of ground-truth data has historically impeded the development of benchmark datasets and objective intercomparisons for machine-learning-based algorithms. Third, the most established existing approaches rely on hand-annotation of plumes and evaluation by domain experts, making their rapid application across large data volumes challenging and costly. Finally, the high societal relevance of these emissions—involving economic losses, health risks, and significant climate forcing—creates a critical trade-off between missing real emissions (false negatives) and mistakenly identifying emissions (false positives). Because unreliable detections could threaten the credibility of remote sensing observations broadly, ensuring precision is essential to keeping satellite data actionable.

The MAPL-EMIT model presented here addresses these challenges by maximizing the information content extracted from EMIT observations. To circumvent the relatively coarse spectral resolution, the model directly utilizes all 285 EMIT spectral bands (except for a few as described in the *SI Appendix*) and uses a vision transformer providing spatial context to jointly retrieve methane enhancement across all pixels within a scene. Analysis of patch embedding weights confirms that the model inherently prioritizes the three primary methane absorption manifolds (*SI Appendix*, Fig. S11). Sensitivity to other bands, such as those near the 700 nm and 2,000 nm bands, may be attributed to water vapor or CO_2_ absorption bands. Furthermore, distributed weights across the remaining spectrum likely facilitate robust background baselining and surface characterization. This comprehensive spectral utilization ensures robust discrimination of true plumes from surface-induced artifacts.

To resolve the historical lack of annotated ground truth, the model was developed using 3.6 million physics-based synthetic plumes superimposed on real EMIT scenes (*Methods*). This approach allows for rigorous optimization and an explicit strategy for balancing detection trade-offs by adjusting plume mask thresholds (*SI Appendix*, Fig. S12). While we optimized thresholds to maximize the F1-score for the 100 to 200 (kg/h)/(m/s) range, the framework is highly adaptable. As detailed in the *SI Appendix*, we provide both model ensemble detection fractions (representing the number of plume detections across overlapping strided inferences) and spectral fit scores for each plume in the released data. This allows end users to dynamically adjust these parameters to suit their specific precision and recall requirements. Taken together, these innovations push the boundaries of emission detection by increasing sensitivity to smaller emissions and enabling the segmentation of overlapping plumes as described below.

### MAPL-EMIT Model Performance.

Evaluating the model against a held-out synthetic test set demonstrates its ability to reliably capture plumes in the 100 to 200 (kg/h)/(m/s) range, with F1 scores, precision, and recall all exceeding 50% for both individual and overlapping plumes. At a nominal wind speed of 2.5 m/s, this corresponds to leak rates of 250 to 500 kg/h, representing an approximate 2 to 4× improvement over existing models (28, 29), which put the 10% probability of detection at around 1,000 kg/h at 2.5 m/s wind speed. Furthermore, the model achieves a recall exceeding 80% for larger emissions above 800 (kg/h)/(m/s) (Fig. 1).

Comparisons with the EMIT L2B product confirm the ability to capture the majority (84%) of highly vetted real-world plumes. Notably on the same set of 1,084 granules included in the comparison to the EMIT L2B plumes, MAPL-EMIT achieved an enhancement SMAPE of 56% when comparing the model-estimated enhancement to the spectral fit enhancement across all the predicted plumes. For context, EMIT L2B plumes obtain an enhancement SMAPE of 117% on the same granules. This reduction in error demonstrates that the enhancements predicted by MAPL-EMIT correspond more closely with expected physical distributions, displaying fewer anomalous high and low-noisy enhancements.

In addition, MAPL-EMIT identifies hundreds of additional plumes in the same granules. While an investigation of all new detections is beyond the scope of this paper, a visual inspection of a subset of the additional identified plumes indicates that many are collocated with plausible methane leak locations (e.g., oil and gas infrastructure; *SI Appendix*, Fig. S5).

Retrievals near landfills and urban-industrial areas often suffer from false positives caused by surface albedo variability and background clutter. Traditional adaptive matched filters are highly sensitive to these artifacts, requiring conservative thresholds or manual review to maintain precision. In contrast, the model achieved a 96% recall across the world’s top-emitting landfills while effectively suppressing surface-induced artifacts. Additionally, evaluations using synthetic artifacts (which mimic the methane spectral signature but lack realistic plume morphology) confirmed that the model effectively isolates true plumes from background noise (*SI Appendix*, Fig. S13). While hallucinations, i.e., model predicting fake plumes over these artifacts, can occur at high artifact intensities, the model generally discards these nonphysical features.

In industrial regions, proximal leaks often coalesce downwind of emission sources. This overlap presents a significant challenge for traditional approaches. We find that MAPL-EMIT is able to disentangle these signals, enabling instance-level segmentation even in complex, multiplume scenarios. Qualitative synthetic results in *SI Appendix*, Fig. S3 and comparisons to the EMIT L2B plumes (e.g., Fig. 2) show successful plume delineation in the presence of overlapping plumes. Evaluation using synthetic plumes suggests that the performance of the model in terms of recall and precision is only marginally impacted by the presence of multiple plumes as shown in Fig. 1.

### Opportunity for Large-Scale Deployment.

MAPL-EMIT provides an end-to-end deep learning framework for the automated detection and quantification of methane enhancements, plume delineation, and source localization from EMIT hyperspectral imagery. The model overcomes many of the limitations of traditional matched-filter algorithms, offering increased sensitivity to low-emission sources and a capability to resolve overlapping plume instances. Results on synthetic and real EMIT observations establish that transformer-based models trained on high-fidelity radiative transfer simulations can generalize effectively to global satellite observations. Integrated enhancement SMAPE statistics across varying emission rate bins (*SI Appendix*, Fig. S2) confirm the framework’s capacity to accurately estimate integrated mass for emission rate quantification.

Taken together, this framework enables methane plume identification across the historical archive of EMIT observations. While performance on future data remains dependent on sensor calibration and degradation over time, a significant advantage of MAPL is its capacity for simple retraining, as it leverages synthetic data instead of relying on expert-annotated labels. The complete set of MAPL-EMIT outputs across the existing EMIT catalog has been made available, as detailed in the *Data, Materials, and Software Availability* section.

This plume database reveals a vast landscape of previously undocumented potential emissions, particularly at the lower end of the intensity spectrum, expanding the utility of global satellite archives to drive mitigation efforts. False positives remain an open challenge, with MAPL-EMIT, like other machine learning models in the literature, showing substantially more false positives than existing expert-vetted plume catalogs such as the EMIT L2B plume complexes. The false positive rate can be reduced by additional filtering based on spectral fit, estimated plume enhancement levels, and/or plume persistence levels as discussed in Results. To build a deeper understanding of the false positive landscape, subsequent research could further investigate scalable and automated methods for identifying false positives. Overall, MAPL-EMIT provides a valuable addition to global methane emissions monitoring by lowering the plausible detection limit in an operationalizable context, even if some post hoc review of model-identified plumes may still be required. Note also that the catalog is not expected to be complete, as indicated by the ~16% of the EMIT L2B plume complexes not captured by the model.

Future research will extend this synthesis pipeline to support multigas and multisatellite retrieval and evolve the architecture toward end-to-end emission rate estimation, enabling direct leak rate emission without requiring post hoc wind integration.

## Methods

MAPL-EMIT was designed to simultaneously delineate methane plumes, quantify their enhancements, and identify sources, directly from full spectrum radiance data. The model was trained on EMIT L1B radiances (30) with injected synthetic methane plumes as outlined in *SI Appendix*, Fig. S14. Below we step through the process of synthetic plume generation, the specific model architecture, model training, evaluation on synthetic plumes and evaluation on real data.

### Dataset Preparation.

EMIT L1B at-sensor radiances (30, 31) were utilized to train and evaluate the model. Using Google Earth Engine, we partitioned the data into nonoverlapping 256 × 256 pixel tiles at the native 60 m spatial resolution. To ensure high data quality, we filtered out tiles with more than 50% cloud cover or spacecraft obstructions, utilizing EMIT L2A surface reflectance masks. Additionally, to prevent the model from overfitting to specific surface or atmospheric conditions, we limited our sampling to a single, randomly selected timestamp for any given spatial location. The resulting dataset consisted of 235 k tiles, which were divided into training (70%), validation (15%), and testing (15%) sets (containing 167 k, 35 k, and 33 k tiles respectively). For synthetic plume validation and test analysis in the rest of the paper, we randomly subsampled 4,096 tiles to optimize evaluation time. This subset provided a statistically significant sample of ~270 million pixels (4,096 × 256 × 256) for robust performance characterization.

To ensure the physical validity of the radiative transfer, we exclude a small number of bands from the EMIT L1B radiance inputs prior to processing. Specifically, we remove seven bands around the order sorting filter, as well as the first three and last three bands to avoid noise, following the discussion in the EMIT Greenhouse Gases Algorithm Theoretical Basis Document (ATBD) (23) and the ISOFIT retrieval algorithm.

### Synthetic Plume Generation.

We developed a large scale synthetic plume dataset using a Lagrangian puff model (32) to simulate methane releases using the hyperparameters listed in *SI Appendix*, Table S1. Plume origins were sampled randomly in clusters to ensure overlapping tails of plumes are present in some cases. Each source produces emissions with random intermittency similar to real releases. The model tracked the 2D movement of individual puffs dictated by a stochastic wind field. Simplex noise (33) was used to simulate turbulence and eddies. Simulations were restricted to 2D because spaceborne instruments measure column-integrated enhancements; this approach significantly accelerated simulation speeds (see examples in *SI Appendix*, Fig. S15). Final concentrations and plume instance masks were derived by aggregating all puffs within the simulation region.

Simulations were performed on a 384 × 384 pixel grid where each pixel is 60 × 60 m to match EMIT’s native spatial resolution. To introduce variation, we generated plumes at 384 × 384 and during training, extracted random 256 × 256 pixel crops, while a deterministic central crop was used for evaluation and testing.

We generated 3.6 million plumes across 240 k tiles, partitioned into training, validation, and testing sets consistent with the EMIT tiles. During training, EMIT tiles are randomly paired with plume tiles, varying by epoch. Evaluation and testing utilize fixed pairings for reproducibility. Each tile contains 15 plumes with corresponding instance masks and origin labels. We randomly select a subset of up to 10 plumes to inject during training for each tile. Every plume was initially simulated with a fixed emission rate of 1 mol/s. This dataset has been publicly released (see *Data, Materials, and Software Availability* section for details) to facilitate further research. Subsequently, these plumes were scaled during radiative transfer to achieve target emission rates in the range of 100 to 10,000 kg/h for training and evaluation. Plume masks were defined using a threshold of 0.00024 ppm-m (corresponds to 0.00001 millimoles/m^2^) on the prescaling 1 mol/s emission rate plumes and smoothed via erosion-dilation filtering to refine boundary edges.

Because there is a general lack of sufficient labeled data, it is not possible to comprehensively evaluate realism of the synthetic plumes against large quantities of real plumes. Instead, the realism (or lack thereof) of the synthetic data becomes evident when the trained model is used with actual EMIT data. If the synthetic data used to train the model had not been realistic, we would not have achieved useful results when applying the resulting model to real EMIT observations. In other words, the realism of the synthetic data is confirmed indirectly through the fact that the resulting model performs well on EMIT observations when evaluated against the real-world benchmarks.

### Plume Injection.

Atmospheric gases exhibit distinct absorption spectra across varying wavelengths, a characteristic fundamental to CH_4_ identification via hyperspectral data. Elevated CH_4_ concentrations increase photon absorption at specific wavelengths. To model these interactions, we employed a line-by-line radiative transfer (34) approach using high-resolution spectral data from the HITRAN database (35). Our calculations accounted for pressure-induced line broadening and thermal induced Doppler effects while incorporating continuum absorptions and emissions from N_2_, O_2_, and H_2_O. This gives a similar transmittance profile at high spectral fidelity as is typically found in band models like MODTRAN (36). Additional details on the transmittance calculations are provided in the *SI Appendix*.

For computational efficiency, and following the majority of matched-filter literature, we precalculated transmittance profiles into a Look-Up Table (LUT), spanning solar and instrument zenith angles as well as CH_4_ concentration (following ref. 37). Water vapor and aerosol variation were not considered in the LUT for simplicity, and we found that model performance was not substantially impacted by this choice (*SI Appendix*, Fig. S16). The LUT was then used to perform radiative transfer following the Beer–Lambert law (38). For simplicity, we ignore scattering effects, making the assumption that atmospheric path reflectance is small in the longer wavelengths where methane absorptions are strongest.

### Deep Learning Framework.

Our deep learning model takes EMIT radiances, angular metadata (solar and satellite zeniths), and crosstrack IDs as input to estimate methane enhancements. The ground truth labels consist of three components per plume: the methane enhancement (in millimoles/m^2^), a binary segmentation mask, and the plume origin.

Simultaneous resolution of all three of these components is a gap in the existing literature (29), largely because without simulation data it is very challenging to have accurate data at large enough scales to train a complex model. MAPL-EMIT’s explicit leveraging of realistic, synthetic data helps overcome this challenge.

### Architecture.

The model utilizes a U-Net-like architecture (39) (*SI Appendix*, Fig. S17) that couples a Swin-v2-S Transformer encoder (40) (~30 M parameters) with a convolutional decoder. The encoder processes all the inputs mentioned above. It progressively downsamples input features, while the decoder reconstructs spatial resolution through a series of upsampling convolutional blocks. Skip connections bridge corresponding stages of the encoder and decoder, enabling the decoder to leverage multiscale features, which is critical for precise spatial localization.

The final decoder feature map feeds into three parallel groups of prediction heads; each group consists of *P* “slot” heads where *P* corresponds to the maximum number of plumes the model can detect per tile, to disentangle individual plumes:

• Enhancement heads: Each head is trained to predict the methane enhancement for a single plume instance.
• Instance mask heads: These mirror the slot structure to generate a binary segmentation mask representing the spatial footprint of each plume.
• Origin heads: These mirror the slot structure to estimate plume origins, where each origin is represented as a binary circle of radius 15 pixels and treated as a binary segmentation task.

For this study, we set P=10, which is sufficient for the typical plume density in the area covered by our 256 × 256 pixel tiles.

### Training.

Synthetic plumes were cropped to 256 × 256 pixels to add variation during training, ensuring that realistic scenarios such as diffused plume tails without visible origins are captured. Plume enhancements were selected by randomly selecting a bin from [100, 200, 400, 800, 1,600, 3,200, 6,400, 12,800] ppm-m and then picking a value uniformly within that bin. These plumes were then injected into the “background” EMIT radiance as outlined above. For evaluation, emission rates were log-uniformly sampled from 100 to 10,000 kg/h to match a realistic real-world distribution of plumes (41). Emission rates were converted to mol/s = kg_per_hour / (3600 × (16.04 / 1000)), where 16.04 is the molar mass of methane, and plume enhancements were scaled linearly to achieve target intensities. 90% of training samples contained plumes as sampled above, but the remaining 10% contained no plumes to ensure that the model is not biased toward always predicting a plume.

The model was trained using a multicomponent loss function where enhancement losses are computed on a sublinear (square root) scale. This transformation allows the model to prioritize lower-intensity enhancements rather than being dominated by high-magnitude plumes. We ablated between linear, square root, and log transformations (*SI Appendix* and *SI Appendix*, Table S2), and square root performed the best. Establishing correspondence between unordered predictions and ground truth plumes requires solving an assignment problem, which we addressed using the Hungarian matching algorithm (42).

[1] $$L_{\delta }\left(y,\hat{y}\right)=\left\{\begin{array}{ccc} \frac{1}{2}{\left(y-\hat{y}\right)}^{2} & \text{for}\left|y-\hat{y}\right|\leq \delta ,\delta \left(\left|y-\hat{y}\right|-\frac{1}{2}\delta \right) & \text{otherwise} \end{array}\right..$$

For a single prediction-ground truth pair $\left(i,j\right)$, the enhancement loss is the Huber loss ($L_{\delta }$) (43) (Eq. **1**) on square-rooted labels (y) and predictions ($\hat{y}$) where δ is the critical threshold, while both the mask and origin losses utilize the binary cross-entropy (BCE) loss. The Hungarian algorithm minimizes the total cost across matches to find the paired slot loss, $L_{\mathrm{slot}}$. Additionally, we applied auxiliary “total enhancement”, “total semantic,” and “total origin semantic” losses to guide the model toward conserving total methane mass.

The final training objective, $L_{\mathrm{final}}$, is a weighted sum of the paired slot loss and the auxiliary total loss.

Let σ be the optimal one-to-one matching (permutation) found by the Hungarian algorithm. The final loss is defined as

[2] $$L_{\text{final}}=\lambda_{\text{slot}}L_{\text{slot}}+\lambda_{\text{total}}L_{\text{total}},$$

Where $L_{\text{slot}}$ is the minimum sum of paired losses across *P* slots defined as

[3] $$\begin{array}{ll} L_{\text{slot}} & =\sum_{i=1}^{P}(\lambda_{\text{enh}}L_{\text{huber}}\left(\sqrt{E_{i}},\sqrt{E_{\sigma \left(i\right)}^{gt}}\right)\\ & \;\;\;+\lambda_{\text{mask}}L_{\text{bce}}\left(M_{i},M_{\sigma \left(i\right)}^{gt}\right)\\ & \;\;\;+\lambda_{\text{origin}}L_{\text{bce}}\left(O_{i},O_{\sigma \left(i\right)}^{gt}\right)). \end{array}$$

The second component $L_{total}$ constrains the aggregated sums for enhancements and the max of instance masks for plume masks (M), and origins (O):

[4] $$\begin{array}{ll} L_{\text{total}} & =\lambda_{\text{e\_total}}L_{\text{huber}}\left(\sqrt{\sum E_{i}},\sqrt{\sum E_{i}^{\text{gt}}}\right)\\ & \;\;\;+\lambda_{\text{m\_total}}L_{\text{bce}}\left(\text{max}\;M_{i},\text{max}\;M_{i}^{\text{gt}}\right)\\ & \;\;\;+\lambda_{\text{o\_total}}L_{\text{bce}}\left(\text{max}\;O_{i},\;\text{max}\;O_{i}^{\text{gt}}\right). \end{array}$$

We selected the weights for each of the loss components to ensure that the scale of all the slot level losses are ~2× the scale of all the top level aggregated losses (*SI Appendix*, Table S3). Additionally, at each slot/top level, we ensure that the losses across heads are roughly at the same scale. We do this after the model stabilizes at ~50 k steps. The final loss weights were set as follows: $\lambda_{\text{e\_total}}=6.0$, $\lambda_{\text{m\_total}}=1.0$, $\lambda_{\text{o\_total}}=19.0$, $\lambda_{\mathrm{enh}}=2.6$, $\lambda_{\mathrm{mask}}=1.0$, and $\lambda_{\mathrm{origin}}=24.0$.

Last, we also upweighted plume pixels by 30.0 on the enhancement losses and 1.0 (no upweight) on the plume mask and origin losses (across both the slot loss and the total loss) to ensure that the model focuses more on plume pixels compared to the background pixels. An upweight sweep ablation is reported in the *SI Appendix*, Table S4.

The model was implemented in Jeo (44) and trained using the AdamW optimizer (45) with parameters $\beta_{1}$ = 0.9, $\beta_{2}$ = 0.999, eps = $1e^{-9}$ and a weight decay of 1e^−9^. We employed a cosine decay learning rate schedule with a linear warmup for the first 1,000 steps to a peak learning rate of 3e^−4^, followed by a decay to a minimum of 0. The training consisted of two stages, the first using a batch size of 128 for 500 k steps and the second using a batch size of 256 for 1 M steps fine-tuning the model from the first stage. All experiments were conducted on Google TPUs (32 chips), with the complete training process taking approximately 96 h.

### Model Inference.

To transition MAPL-EMIT from fixed-window patch predictions (256 × 256 pixels, ~234 km^2^) to EMIT granule level predictions (~5,700 km^2^), we developed a large-scale inference pipeline that operates at a granule level designed to produce two distinct outputs: a methane enhancement raster and a set of plume instances, including individual plume enhancements.

To generate the enhancement raster, the pipeline processed full granules using overlapping spatial strides of 64 pixels. Multislot predicted enhancements were summed and reconstructed using a Hanning window function (46), which seamlessly merged overlapping tiles into a continuous raster by weighting central pixel predictions to eliminate edge discontinuities. To produce the plume instance collection, the pipeline resolved redundant detections caused by the overlapping strides through a custom spatial clustering algorithm inspired by DBSCAN (47). This process clustered candidate plumes into distinct entities by jointly evaluating spatial proximity of predicted plume origins and plume-head enhancement correlation. Finally, all isolated candidates were subjected to the physics-based spectral vetting pipeline (see *Spectral Fit Comparison* section below) to produce spectral fit metrics. A description of the entire pipeline is provided in the *SI Appendix*.

### Model Evaluation.

#### Synthetic evaluation.

We evaluated the model’s tile-level performance across three core tasks: enhancement quantification, plume detection, and origin estimation. A systematic evaluation on a held-out synthetic test set was essential, as it provided precise ground-truth labels often unavailable in real-world observations. By stratifying these results across simulated wind and emission conditions, we quantified sensitivity limits and established operating thresholds for large-scale monitoring.

Performance was assessed using distinct metrics for each task:

1. Enhancement Quantification: We computed label normalized Root Mean Squared Error (RMSE) (6, 47) and Symmetric Mean Absolute Percentage Error (SMAPE) (48). These metrics were averaged across all *P* output slots to get the final numbers.
2. Plume Delineation: We calculated precision, recall, F1 score, and integrated enhancement SMAPE across plume instances. Precision–Recall (PR) curves were generated to identify the optimal operating threshold based on predicted mask probabilities.
3. Source Localization: We computed the precision, recall, and mean origin euclidean distance for true positives in meters.

All enhancement metrics were stratified by grouping pixels into buckets of label enhancements [0, 5, 25, 50, 125, 250, 500, 1,000, 2,500, 25,000] ppm-m. Plume detection metrics were stratified by grouping ground-truth plumes into buckets of emission rate divided by wind speed (ERBWS) [50, 100, 200, 400, 800, 1,600, 3,200], which correspond to approximately [125, 250, 500, 1,000, 2,000, 4,000, 8,000] kg/h at 2.5 m/s wind speeds. We evaluated ERBWS bins because emissions and wind speeds exhibit an inverse linear sensitivity relationship, facilitating consistent performance tracking across distinct categories. The validation and test splits utilized a log-uniform emission rate distribution because it gives us a roughly similar number of plumes in each logarithmic ERBWS bucket during evaluation.

We selected the final model, operating plume, and origin probability thresholds by maximizing the plume instance F1 score for the 100 to 200 ERBWS bucket on the validation split (note that in *SI Appendix*, Fig. S12, 0.3 is the optimal threshold that maximizes the F1 score but all our parameter selection was done on the validation split to avoid overfitting, where 0.4 turned out to be the optimal threshold). This range (250 to 500 kg/h at 2.5 m/s) represents our primary focus, as it encompasses a significant fraction of real-world methane leaks following a heavy-tailed distribution (41, 50).

To derive discrete predicted origins from the model’s origin probability maps, we utilized a threshold-based weighted centroid approach. For each plume slot, we identify all pixels where the predicted probability exceeds a threshold of 0.3 (this threshold gave us the best metrics on the synthetic validation split). The final estimated origin coordinate (x,y) is computed as the weighted mean of these pixel locations, using the predicted probabilities $P_{i}$ as the weights (Eq. **5**).

[5] $$\hat{x}=\frac{\sum P_{i}x_{i}}{\sum P_{i}},\;\hat{y}=\frac{\sum P_{i}y_{i}}{\sum P_{i}}.$$

All the metrics are calculated after aligning predicted plumes, origins, and enhancements with ground-truth labels via Hungarian matching of semantic masks. For the plume detection metrics, a prediction was classified as a True Positive (TP) if its IoU with a ground-truth mask exceeded 0.25. Predictions failing this threshold were counted as False Positives (FP), while unmatched ground-truth plumes were False Negatives (FN). Similarly, for origin estimation, a prediction was classified as a True Positive if it was within 600 m in terms of euclidean distance. The model was trained to produce empty outputs for detection and origin slots that do not correspond to a ground-truth plume.

*SI Appendix*, Fig. S12 illustrates performance trade-offs based on the plume probability threshold, depicting the PR curve for the 100 to 200 ERBWS bucket, the balance between true positives and false positives, and the resulting precision and recall curves. These curves demonstrate the model’s ability to maintain high precision via a plume probability threshold adjustment, which is critical for operational monitoring where false alarms must be minimized.

#### Spectral fit comparison.

To verify the model’s real-world performance, we employed spectral fit comparisons (24) as an independent verification metric across all real-world evaluations. This method serves to verify the physical validity of detected plumes by comparing the observed radiance against modeled spectra (*SI Appendix*, Figs. S18–S20).

As described in ref. 24, the methodology proceeds as follows. We define in-plume pixels by taking the model predicted mask, excluding the top 1% of model predicted enhancements, and sampling 3 × 3 regions around the top 30 remaining pixels. Due to spatial overlap, this typically yields 100 to 150 unique pixels. To select out-of-plume (background) pixels, we expand the plume mask by 200 pixels, remove any other model predicted plumes in the granule, and exclude pixels with enhancements >30 ppm-m. We then use Hungarian matching on nonmethane absorption bands to pair each in-plume pixel with its closest background match. After computing mean radiance ratios for the in-plume and out-of-plume pixels individually, we apply a least-squares optimization to fit parameters, including a fitted enhancement (fit_enh). We then compare the mean of the model’s predicted enhancements across the in-plume pixels (obs_enh), against fit_enh. Spectral fit scores are assessed using correlation error ($D_{\mathrm{cor}}$) and normalized error ($D_{\mathrm{norm}}$) obtained by comparing the fitted and observed transmittance ratios.

A correlation error ($D_{\mathrm{cor}}$) below 0.4 and a normalized error ($D_{\mathrm{norm}}$) below 0.5 were found by ref. 24 to typically identify valid methane plumes. While high error values of $D_{\mathrm{norm}}$ above 0.8 generally suggest false positives, detections with elevated $D_{\mathrm{cor}}$ or $D_{\mathrm{norm}}$ were still considered legitimate if they coincided with high spectral fit estimated methane enhancements (fit_enh) exceeding 100 ppm-m.

This spectral analysis is particularly critical for low-intensity plumes that lack traditional ground-truth labels. We therefore use these thresholds to identify potential true plumes in the 20,000 granules used in the false positive rate assessment and to evaluate the additional plumes identified in the granules used in the comparison to the NASA EMIT L2B plume complexes.

#### Real world evaluation.

To evaluate the model’s generalizability from synthetic training to real-world observations, we compiled a diverse suite of verification and validation datasets. While the inherent scarcity of high-resolution ground-truth data often constrains rigorous quantitative assessments of sensitivity and precision, we leveraged a diversity of available observational benchmarks to ensure a robust performance evaluation. We compare against the EMIT L2B plume complexes as described in the main results, and perform several additional checks as outlined below.

#### High-resolution aerial observations.

To evaluate model performance against high-fidelity aerial sources, we utilized observations from the Airborne Visible/Infrared Imaging Spectrometer-3 (AVIRIS-3) (51) and the Global Airborne Observatory (GAO) (20). These sensors provide superior spatial resolution and SNR compared to spaceborne instruments. We specifically leveraged methane enhancements from these aerial datasets in regions with coincident EMIT overpasses. By comparing MAPL-EMIT’s predicted plumes and enhancements against the L2B methane products from AVIRIS-3 and GAO, we established a benchmark for sensitivity and false-positive suppression in real-world environments.

#### Controlled release experiments.

To validate the model’s detection sensitivity and enhancement quantification accuracy, we utilized data from the Stanford controlled methane release experiments conducted August 1, 2024, to December 31, 2025 (27). These experiments were conducted near a dedicated field site in Arizona ($32.813^{\circ },-111.796^{\circ }$). We specifically analyzed five releases from the Stanford Phase 2 and two releases from the Phase 4 campaigns, allowing for a rigorous assessment of the model’s ability to recover plumes from low-intensity, short-duration releases.

#### Top-emitting landfills.

To validate the model’s performance in complex environments, we evaluated its recall across 25 of the highest-emitting municipal solid waste landfills globally (sampling one random EMIT timestamp at each location). These sites were selected based on a recent hyperspectral survey that identified major methane superemitters using EMIT and other high-resolution sensors (19). Landfills represent a challenging retrieval domain due to high surface heterogeneity and variable albedo, which often induce artifacts in traditional physics-based algorithms. By benchmarking against these established emitters, we assessed the model’s ability to maintain high detection sensitivity and suppress false positives in terrains where background clutter typically limits automated monitoring.

## Supplementary Material

Appendix 01 (PDF)

## Data Availability

The datasets used during this study are made available to support reproducibility and further research. The dataset from NASA [EMIT L1B (52), L2A (53), L2B CH4 ENH (54), L2B CH4 PLM (55), and AVIRIS-3 (56)] are available from the NASA catalog at https://www.earthdata.nasa.gov/data/catalog. The Global Airborne Observatory (GAO) (57) data can be accessed at https://data.carbonmapper.org. To facilitate public access and ensure reproducible research, we release the synthetic plumes (58) that were used to train our model and the fully trained model (59) on the Kaggle platform with the supporting code on GitHub (60). To enable large-scale analysis, we release the model inference outputs across the full EMIT data record as Google Earth Engine image collections, comprising per-granule total enhancement rasters (61) and per-plume instances (62) with segmented methane enhancements for every detection. We also provide an Earth Engine App (63) to visualize the plumes and enhancements via an interactive UI interface.

## References

[1] P. Forster , “Short-lived climate forcers” in V. Masson-Delmotte , Eds., Climate Change 2021: The Physical Science Basis. Contribution of Working Group I to the Sixth Assessment Report of the Intergovernmental Panel on Climate Change (Cambridge University Press, 2021).

[2] M. Ashfold , Global Methane Pledge: A Review of Data, Policy and Transparency in Reducing Methane Emissions in Malaysia (ISEAS–Yusof Ishak Institute, 2023), pp. vii–viii.

[3] International Energy Agency, Global Methane Tracker 2025. IEA (2025). https://www.iea.org/reports/global-methane-tracker-2025.

[4] B. J. Schuit , Automated detection and monitoring of methane super-emitters using satellite data. Atmos. Chem. Phys. **23**, 9071–9098 (2023).

[5] E. Dowd , First validation of high-resolution satellite-derived methane emissions from an active gas leak in the UK. Atmos. Meas. Tech. **17**, 1599–1615 (2024).

[6] B. Rouet-Leduc, C. Hulbert, Automatic detection of methane emissions in multispectral satellite imagery using a vision transformer. Nat. Commun. **15**, 3801 (2024). 38744827 10.1038/s41467-024-47754-y PMC11094139

[7] A. K. Thorpe , Attribution of individual methane and carbon dioxide emission sources using EMIT observations from space. Sci. Adv. **17**, eadh2391 (2023). 10.1126/sciadv.adh2391 PMC10656068 37976355

[8] D. Manolakis, R. Lockwood, T. Cooley, J. Jacobson, Is the matched filter good enough for hyperspectral target detection? IEEE Trans. Geosci. Remote Sens. **52**, 4216–4229 (2014).

[9] D. R. Thompson , Real-time remote detection and measurement for airborne imaging spectroscopy: A case study with methane. Atmos. Measure. Techniq. **8**, 4383–4397 (2015).

[10] A. K. Thorpe , High resolution mapping of methane emissions from marine and terrestrial sources using a Cluster-Tuned Matched Filter technique and imaging spectrometry. Remote Sens. Environ. **134**, 305–318 (2013).

[11] R. Duren , The carbon mapper emissions monitoring system. Atmos. Measure. Techniq. **18**, 6933–6958 (2025).

[12] V. Růžička , Semantic segmentation of methane plumes with hyperspectral machine learning models. Sci. Rep. **13**, 19999 (2023). 37978332 10.1038/s41598-023-44918-6 PMC10656523

[13] R. W. Coleman , Identifying and quantifying greenhouse gas emissions with the AVIRIS-3 airborne imaging spectrometer. Remote Sens. Environ. **332**, 115058 (2026).

[14] D. H. Cusworth , Potential of next-generation imaging spectrometers to detect and quantify methane point sources from space. Atmos. Meas. Tech. **12**, 5655–5668 (2019).

[15] T. Ehret , Global tracking and quantification of oil and gas methane emissions from recurrent sentinel-2 imagery. Environ. Sci. Technol. **56**, 10517–10529 (2022), 10.1021/acs.est.1c08575. 35797726

[16] A. Vaughan , CH4Net: A deep learning model for monitoring methane super-emitters with Sentinel-2 imagery. Atmos. Meas. Tech. **17**, 2583–2593 (2024).

[17] J. Gorroño, D. J. Varon, I. Irakulis-Loitxate, L. Guanter, Understanding the potential of Sentinel-2 for monitoring methane point emissions. Atmos. Meas. Tech. **16**, 89–107 (2023).

[18] A. Thorpe , EMIT L2B estimated methane plume complexes 60 m V002 [Dataset]. NASA Land Processes Distributed Active Archive Center (2026). 10.5067/EMIT/EMITL2BCH4PLM.002.

[19] X. Zhang , Global identification of solid waste methane super emitters using hyperspectral satellites. Environ. Sci. Technol. **59**, 18134–18145 (2025). 40827757 10.1021/acs.est.4c14196 PMC12409875

[20] A. K. Ayasse , Methane remote sensing and emission quantification of offshore shallow water oil and gas platforms in the Gulf of Mexico. Environ. Res. Lett. **17**, 084039 (2022).

[21] E. D. Sherwin , Single-blind test of nine methane-sensing satellite systems from three continents. Atmos. Meas. Tech. **17**, 765–782 (2024).

[22] J. E. Fahlen , Sensitivity and uncertainty in matched-filter-based gas detection with imaging spectroscopy. IEEE Trans. Geosci. Remote Sens. **62**, pp. 1–10 (2024).

[23] P. G. Brodrick, A. K. Thorpe, D. R. Thompson, R. O. Green, “EMIT L2B greenhouse gas algorithm theoretical basis document (ATBD)” (Jet Propulsion Laboratory, California Institute of Technology, 2025).

[24] C. Xiang , Identification of false methane plumes for orbital imaging spectrometers: A case study with EMIT. Remote Sens. Environ. **328**, 114860 (2025).

[25] Z. N. Armstrong, H.-N. Kostis, A. Thorpe, NASA Scientific Visualization Studio. https://svs.gsfc.nasa.gov/5272. Accessed 20 October 2025.

[26] AVIRIS-3 L2B Greenhouse Gas Enhancements, Facility instrument collection. NASA Earthdata; Earthdata. https://www.earthdata.nasa.gov/data/catalog/ornl-cloud-av3-l2b-ghg-2358-1. Accessed 9 November 2025.

[27] F. Reuland, T. Adams, K. Galvin, E. A. Kort, A. R. Brandt, Large-scale controlled methane releases for satellite-based detection and emission quantification of point-sources. Stanford Digital Repository. (2026). 10.25740/qh001qt3946.

[28] A. K. Ayasse , Probability of detection and multi-sensor persistence of methane emissions from coincident airborne and satellite observations. Environ. Sci. Technol. **58**, 21536–21544 (2024). 39587769 10.1021/acs.est.4c06702 PMC11636213

[29] E. Tiemann , Machine learning for methane detection and quantification from space: A survey. IEEE Geosci. Remote Sens. Magaz. **99**, 2–28 (2025).

[30] R. Green, EMIT L1B at-sensor calibrated radiance and geolocation data 60 m V001 [Dataset]. NASA Land Processes Distributed Active Archive Center (2022). 10.5067/EMIT/EMITL1BRAD.001.

[31] D. R. Thompson , On-orbit calibration and performance of the EMIT imaging spectrometer. Remote Sens. Environ. **303**, 113986 (2024).

[32] P. Hurley, PARTPUFFA Lagrangian particle-puff approach for plume dispersion modeling applications. J. Appl. Meteorol. **33**, 285–294 (1994).

[33] K. Perlin, Simplex noise. (2001). https://userpages.cs.umbc.edu/olano/s2002c36/ch02.pdf.

[34] S. A. Clough , Atmospheric radiative transfer modeling: A summary of the AER codes. J. Quant. Spectrosc. Radiat. Transfer **91**, 233–244 (2005).

[35] I. E. Gordon , The HITRAN2020 molecular spectroscopic database. J. Quantit. Spectroscopy Radiat. Transf. **277**, 107949 (2022).

[36] A. Berk , “MODTRAN® 6: A major upgrade of the MODTRAN® radiative transfer code” in 2014 6th Workshop on Hyperspectral Image and Signal Processing: Evolution in Remote Sensing (WHISPERS, 2014), pp. 1–4.

[37] A. K. Thorpe, C. Frankenberg, D. A. Roberts, Retrieval techniques for airborne imaging of methane concentrations using high spatial and moderate spectral resolution: Application to AVIRIS. Atmos. Meas. Tech. **7**, 491–506 (2014).

[38] D. F. Swinehart, The Beer-Lambert Law. J. Chem. Educ. **39**, 333 (1962).

[39] O. Ronneberger, P. Fischer, T. Brox, “U-Net: Convolutional networks for biomedical image segmentation” in *Medical Image Computing and Computer-Assisted Intervention–MICCAI 2015*, N. Navab, J. Hornegger, W. Wells, A. Frangi, Eds. (Springer, Cham., 2015), vol. **9351**, pp. 234–241.

[40] Z. Liu , “Swin transformer V2: Scaling up capacity and resolution” in IEEE/CVF Conference on Computer Vision and Pattern Recognition, CVPR (2022), pp. 11999–12009.

[41] D. J. Jacob , Quantifying methane emissions from the global scale down to point sources using satellite observations of atmospheric methane. Atmos. Chem. Phys. **22**, 9617–9646 (2022).

[42] R. Stewart, M. Andriluka, A. Y. Ng, “End-to-end people detection in crowded scenes” in 2016 IEEE Conference on Computer Vision and Pattern Recognition (CVPR) (Las Vegas, NV, USA, 2016), June, 10.1109/cvpr.2016.255.

[43] P. J. Huber, Robust estimation of a location parameter. Ann. Math. Stat. **35**, 73–101 (1964).

[44] Team J, JEO: Model training and inference for geospatial remote sensing and Earth Observation in JAX. GitHub. https://github.com/google-deepmind/jeo. Accessed 15 November 2024.

[45] I. Loshchilov, F. Hutter, Decoupled weight decay regularization. (2017). http://arxiv.org/abs/1711.05101.

[46] F. J. Harris, On the use of windows for harmonic analysis with the discrete Fourier transform. Proc. IEEE **66**, 51–83 (1978).

[47] M. Ester, H.-P. Kriegel, J. Sander, X. Xu, “A density-based algorithm for discovering clusters in large spatial databases with noise” in Proceedings of the Second International Conference on Knowledge Discovery and Data Mining, KDD-96 (1996), pp. 226–231.

[48] L. Guanter , Mapping methane point emissions with the PRISMA spaceborne imaging spectrometer. Remote Sens. Environ. **265**, 112671 (2021).

[49] S. Makridakis, Accuracy measures: Theoretical and practical concerns. Int. J. Forecast. **9**, 527–529 (1993).

[50] R. M. Duren , California’s methane super-emitters. Nature **575**, 180–184 (2019). 31695210 10.1038/s41586-019-1720-3

[51] A. M. Chlus , AVIRIS-3 L2B greenhouse gas enhancements, facility instrument collection. ORNL Distributed Active Archive Center. 10.3334/ORNLDAAC/2358. Accessed 9 November 2025.

[52] R. O. Green , EMIT L1B At-Sensor Calibrated Radiance and Geolocation Data 60 m v001. NASA EOSDIS Land Processes DAAC. (2024). 10.5067/EMIT/EMITL1BRAD.001. Accessed 1 May 2024.

[53] R. O. Green , EMIT L2A Estimated Surface Reflectance and Uncertainty 60 m v001. NASA EOSDIS Land Processes DAAC. 10.5067/EMIT/EMITL2ARFL.001. Accessed 1 May 2024.

[54] A. Thorpe , EMIT L2B Methane Enhancement Data 60 m v002. NASA EOSDIS Land Processes DAAC. 10.5067/EMIT/EMITL2BCH4ENH.002. Accessed 1 May 2024.

[55] A. Thorpe , EMIT L2B Estimated Methane Plume Complexes 60 m v002. NASA EOSDIS Land Processes DAAC. 10.5067/EMIT/EMITL2BCH4PLM.002. Accessed 1 May 2024.

[56] A. M. Chlus , AVIRIS-3 L2B Greenhouse Gas Enhancements, Facility Instrument Collection, V1. NASA EOSDIS ORNL DAAC. 10.3334/ORNLDAAC/2358. Accessed 15 June 2025.

[57] G. P. Asner, Carbon Mapper Team, Global Airborne Observatory (GAO) High-Resolution Greenhouse Gas Emissions Data. Carbon Mapper. https://data.carbonmapper.org. Accessed 23 August 2025.

[58] V. V. Batchu , Synthetic puff-based plumes dataset. Kaggle. 10.34740/kaggle/ds/9284528. Deposited 5 August 2026.

[59] V. V. Batchu , MAPL-EMIT Model for Global Methane Plume Detection. Kaggle. 10.34740/kaggle/m/575099. Deposited 20 July 2026.

[60] A. Wilson , MAPL codebase. Github. https://github.com/google-research/mapl. Deposited 14 August 2026.

[61] V. V. Batchu , MAPL-EMIT Methane Enhancements. Earth Engine. https://developers.google.com/earth-engine/datasets/catalog/projects_nature-trace_assets_ghg_emit_mapl_emit_enhancements_v1_0. Deposited 14 August 2026.

[62] V. V. Batchu MAPL-EMIT Methane Plumes. Earth Engine. https://developers.google.com/earth-engine/datasets/catalog/projects_nature-trace_assets_ghg_emit_mapl_emit_plumes_v1_0. Deposited 14 August 2026.

[63] V. V. Batchu , MAPL-EMIT Earth Engine App. Earth Engine App. https://nature-trace.projects.earthengine.app/view/mapl-emit. Deposited 15 August 2026.

