# Supplementary material for: Global monitoring of methane point sources using deep learning on hyperspectral radiance measurements from EMIT
Source: Proc Natl Acad Sci U S A. 2026 Sep 1;123(36):e2612145123. doi: 10.1073/pnas.2612145123 (PMC13552665; doi:10.1073/pnas.2612145123)
Supplement: Supplementary file 1 [file pnas.2612145123.sapp.pdf]

PNAS

Supporting Information for

Global monitoring of methane point sources using deep learning on hyperspectral radiance measurements from EMIT

Vishal V. Batchu*[1], Michelangelo Conserva*[1], Alex Wilson*[1], Anna M. Michalak[1,2], Varun Gulshan[1], Philip G. Brodrick[3], Andrew K. Thorpe[3], Christopher V. Arsdale[1]

[1]Google Research, [2]Carnegie Institution for Science, [3]Jet Propulsion Laboratory, California Institute of Technology

Vishal V. Batchu
Email: vishalbatchu@google.com

This PDF file includes:

- Supporting text
- Figures S1 to S23
- Tables S1 to S4
- SI References

Supporting Information Text

Synthetic Data Generation

Transmittance Calculations. We acquired atmospheric transmittances at a spectral resolution of 0.00025 µm over a range of 0.38 to 2.5 µm for a US standard atmosphere (U.S. Standard Atmosphere, 1976, 1976). These data were modeled using SpectralCalc (Gordley et al., 1994) with a nadir-viewing geometry and an observer height of 600 km, hereafter referred to as "standard atmosphere transmittances."

A synthetic atmosphere was subsequently generated by modifying the standard US atmosphere to include a localized methane enhancement. We added 1 ppm of methane from the Earth's surface through the initial 100m of the atmospheric column, followed by a linear decay to 0 ppm over the subsequent 100m. This configuration represents a constant vertical profile of methane release used across all simulations, contributing a total vertical column concentration of 150 ppm-m visualized in Figure S21. While sampling varied methane addition profiles would be ideal, we selected this representative atmosphere to maintain computational scalability. Because the total methane mass added remained fixed, minor variations in the vertical distribution result in negligible differences in the column-integrated absorption signal measured by the instrument. Transmittances were then obtained for this modified atmosphere, hereafter termed "methane transmittances."

Spectral Responses. Laboratory-calibrated spectral response functions (SRFs) for the EMIT bands are accurately approximated by Gaussian distributions (Thompson et al., 2024). For modeling simplicity, we treated each band's SRF as a Gaussian centered at the band mean wavelength. The standard deviation was derived directly from the measured Full Width at Half Maximum for each respective band.

Lookup table based on Beer-Lambert absorption. The transmittance of an atmosphere containing *x* ppm-m of added methane at a given wavelength (λ) is derived by linearizing the Beer-Lambert law. This formulation assumes that the differential absorption remains non-saturating within the modeled concentration range. Because the methane levels utilized in this study did not lead to significant saturation of the absorption lines, the added concentration was applied directly as an exponent (Eqn S1).

$$t^{x}_{\mathrm{methane}}(\lambda) = t_{\mathrm{std}}(\lambda) \cdot \left(\frac{t^{150}_{\mathrm{methane}}(\lambda)}{t_{\mathrm{std}}(\lambda)} \right)^{\frac{x}{150}} \quad \text{(S1)}$$

To account for the total photon path, we adjusted the transmittance by a path length multiplier determined by the solar and satellite zenith angles (Eqn S2). Since SpectralCalc data assumed a single vertical pass of the atmosphere, we performed this adjustment using the same exponential scaling logic (Equation S3) for both CH_4 and standard atmosphere transmittances (t_{std}) as well.

$$\mathrm{path\_length\_multiplier} = \frac{1}{\cos(\theta_{\mathrm{solar}})} + \frac{1}{\cos(\theta_{\mathrm{satellite}})} \quad \text{(S2)}$$

$$t^{x,\mathrm{path\_length\_multiplier}}_{\mathrm{methane}}(\lambda) = t_{\mathrm{std}}(\lambda) \cdot \left(\frac{t^{150}_{\mathrm{methane}}(\lambda)}{t_{\mathrm{std}}(\lambda)} \right)^{\frac{x \cdot \mathrm{path\_length\_multiplier}}{150}} \quad \text{(S3)}$$

We populated a Look-Up Table (LUT) with transmittance ratio multipliers for all combinations of methane concentrations and path length multipliers. This pre-computation was executed once due to its high spectral resolution and computational cost (Eqns S4, S5 and S6).

$$\text{methane\_concentrations} = \{0, 100, \ldots, 900\} \cup \{1000, 2000, \ldots, 9000\} \cup \{100000\} \quad \text{(S4)}$$

$$\text{path\_length\_multipliers} = \{1, 2, 3, 4, 5, 100\} \quad \text{(S5)}$$

$$\begin{aligned}
&\textbf{for each } conc \in \text{methane_concentrations}: \\
&\quad \textbf{for each } \text{path_multiplier} \in \text{path_length_multipliers}: \\
&\qquad LUT(conc, path_length_multiplier) = \log \frac{\sum_{\lambda \in \mathcal{L}} \left[\text{SRF}(\lambda) \cdot \left(t^{x}_{\text{methane}}(\lambda) \right)^{path_length_multiplier} \right]}{\sum_{\lambda \in \mathcal{L}} \left[\text{SRF}(\lambda) \cdot (t_{\text{std}}(\lambda))^{path_length_multiplier} \right]}
\end{aligned} \quad \text{(S6)}$$

During data generation, bounding entries were identified via a LUT search, followed by bilinear interpolation in log-space (Eqn S7). Log-space interpolation was essential to maintaining accuracy because concentration and path length factors remain exponential even after spectral band averaging. The final synthetic radiance was then derived (Eqn S8) using the multiplicative factor computed in Eqn S7.

$$R_{\text{final}}(\text{conc}, \text{path\_length\_multiplier}) = \exp(\text{bilinearInterp}(\log(\text{LUT}))) \quad \text{(S7)}$$
$$L_{\text{syn}}(x) = L_{\text{std}} \cdot R_{\text{final}}(conc, \text{path\_length\_multiplier}) \quad \text{(S8)}$$

Our radiative transfer assumed a constant methane concentration along the photon path. While an oblique viewing geometry (maximal angles of ~45 with methane altitudes up to 200 m) result in a path integral spanning approximately 3.33 pixels, we did not apply path-integrated mixing as the majority of our synthetic plumes exceeded this 3.33 pixel scale and this only introduced a marginal inaccuracy.

To simplify the radiative transfer model, aerosols and water vapor were excluded from the retrieval framework. Sensitivity analyses in Figure S16 where the SMAPE of the integrated enhancements between model detected plumes and NASA plumes indicate a lack of significant correlation between retrieval error (SMAPE) and either water vapor concentration or AOD 550. This analysis was conducted on all plumes detected by MAPL-EMIT that also appear in the NASA L2B plume complexes catalog across the subset of tiles used in the intercomparison (see Main Text). For these instances, we used the NASA L2B plume mask as the ground-truth label for the integration process. The stability of the binned medians (and 25, 75 percentile ranges) across these atmospheric variables suggests that the model is robust to these factors within the observed ranges, justifying their omission to improve computational efficiency.

Input normalization. We implement a two-stage normalization pipeline to standardize EMIT L1B radiances prior to passing them as inputs to the model.

1. We perform a solar spectrum normalization to remove the dominant spectral shape of the solar irradiance (Planck, n.d.). We compute the theoretical spectral radiance of the Sun, modeled as a blackbody at T=5778 K using Planck's Law. This high-resolution blackbody curve is convolved with the specific Spectral Response Function (SRF) for each EMIT band to derive band-specific solar irradiance factors. The input radiances are divided by these factors (scaled by an empirical constant = 4.0 / 1e7 to get the scale of the radiances to be within a rough range of 0-1), effectively converting the signal into a pseudo-reflectance space.
2. We apply a cross-track radiometric correction to mitigate detector-level striping and systematic illumination artifacts across the swath. We pre-compute the means for every band at every cross-track position across all the EMIT granules. For a given pixel, the radiance is adjusted by multiplying it by the ratio of the global mean radiance for that band (averaged across all

crosstrack positions) to the mean radiance for that band for the specific crosstrack ID of the pixel, thereby correcting for multiplicative striping artifacts. Figure S22 shows the difference this makes during inference.

Model architecture. A Swin Transformer encoder progressively downsamples the input radiance data, while a parallel convolutional decoder upsamples the features to reconstruct the tile. Skip connections link the encoder and decoder at corresponding scales to preserve multi-scale spatial information. The final decoder feature map is passed to the prediction heads (shown as "Slot linear projections") to generate multiple output slots for enhancement predictions, plume masks and plume origin masks (Figure S17).

Ablations

All ablations were performed on stage 1 of training over 500k steps using a batch size of 128. Post ablations, the best hyperparameters were selected before training the final model in a two stage manner as mentioned in the Methods section.

Output scaling. To ensure the model effectively learns from the full dynamic range of emissions, we investigated different scaling strategies designed to prioritize the accurate retrieval of lower-intensity plumes while maintaining performance on larger plumes. Both square root and log1p perform much better than no scaling (linear) but square root ended up performing marginally better.

Plume region upweighting. To ensure the model deals with the imbalance of plume vs non plume pixels effectively, we explore various plume region upweights for the enhancement loss and the instance segmentation losses. An upweight of 30x for plume pixels on the enhancement loss and no upweight for the instance segmentation losses worked best.

Loss ablations. To regularize the model's slot predictions, we added additional aggregate losses to the enhancement, plume and origin predictions. These help ensure that the amount of methane is captured correctly in a scene. We see that both the aggregate enhancement and segmentation losses help improve model performance.

Synthetic evaluation failure cases.

Failures primarily occur in complex scenarios where the target signal is obscured: plume overlap in high-density areas causes localization errors, while temporal intermittency and fragmented plumes lead to reduced recall (Figure S23). Additionally, low-albedo surfaces (e.g., water) result in poor signal-to-noise ratios, often masking enhancements.

Granule level inference

Overview. To facilitate global-scale methane retrieval, we extend the MAPL-EMIT framework to process contiguous EMIT granules. While the core model operates on fixed 256x256 pixel spatial windows, real-world super-emitter emissions often exceed this spatial extent. To mitigate boundary truncation, suppress duplicate detections, and seamlessly aggregate predictions across large geographical areas, we employ a strided inference and spatial clustering methodology. This also functions to create an ensemble of model predictions, giving us an additional metric of model confidence (the percentage of times a given plume appears in each prediction ensemble).

Strided inference and spatial reconstruction. To mitigate the artificial bisection of spatially extended plumes, the framework processes full EMIT granules using overlapping spatial strides. The model iteratively evaluates 256x256 pixel tiles with a spatial stride of 64 pixels, yielding a 75% overlap between adjacent windows. While this strided approach ensures complete morphological capture, it inherently generates redundant candidate predictions for individual physical plumes across adjacent spatial windows.

To resolve boundary artifacts and reconstruct continuous emission fields, overlapping predictions are aggregated using a spatial Hanning window (L2 distance averaging). This approach assigns higher weights to predictions near the center of each inference chunk, thereby minimizing edge-effects and seamlessly merging overlapping probability maps into a continuous global raster.

Candidate consolidation and denoising. While strided inference reconstructs the total spatial extent of the plumes, it produces multiple overlapping instance candidates for a single physical source. To consolidate these multiple detections, we introduce a feature-level and spatial clustering algorithm inspired by DBSCAN. Standard spatial clustering relies solely on proximity, which can erroneously merge distinct, proximal emission sources common in dense industrial regions. To preserve instance separation, our algorithm enforces two matching criteria for candidate consolidation:

1. Spatial Proximity: The Euclidean distance between the predicted plume origins must not exceed a predefined threshold of 1.5 km.
2. Enhancement Correlation: For candidate pairs meeting the proximity constraint, local enhancement patches centered on their respective origins are extracted and aligned. The Pearson correlation coefficient is computed across overlapping pixels, requiring a correlation exceeding 0.955 for the candidates to be classified as identical.

Consolidated candidates are aggregated over a joint bounding box using a regularized weighted mean. Finally, a connected components analysis is applied to the thresholded binary masks to remove high-frequency noise, discarding components smaller than 36 pixels. This yields discrete, vectorized plume polygons suitable for geospatial analysis. We filter out plumes with an inference cluster size > 13. Additionally, we provide all the plume statistics along with each plume to allow end users to dynamically select thresholds that align with their specific sensitivity and precision requirements.

Hyperparameter optimization. Hyperparameters were chosen in two stages. In the first stage, model training hyperparameters were tuned on synthetic granules where exact precision and recall could be calculated and optimised. In the second stage, inference hyperparameters were tuned on real-world data. This real-world data included both the EMIT L2B plumes dataset (which only allowed measuring recall as it has no negative examples) and the non-emission granules where experts expect a minimal number of plumes. The refinement focused on finding a good balance of maximizing recall on the NASA plumes while reducing the number of unexpected detections in the plume-free regions.

Figures

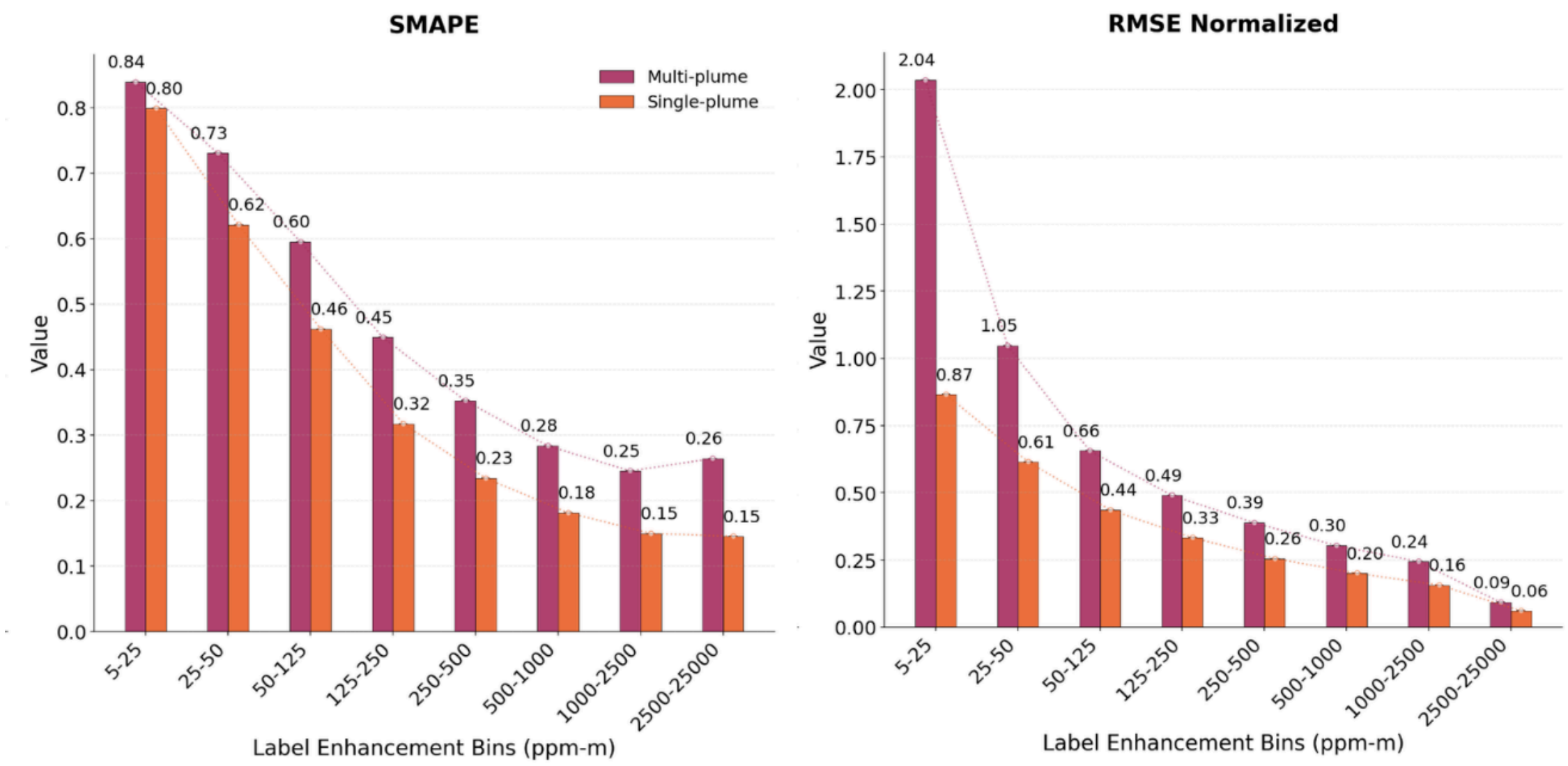

Figure S1: MAPL-EMIT demonstrates robust quantification of methane enhancements across a wide dynamic range. Performance metrics, including SMAPE and RMSE (normalized by mean bin enhancement), are stratified by label enhancement buckets to characterize model sensitivity from 250 to 25,000 ppm-m. Slot-level evaluation on the synthetic test split shows high accuracy from the 50-125 ppm-m bucket onwards.

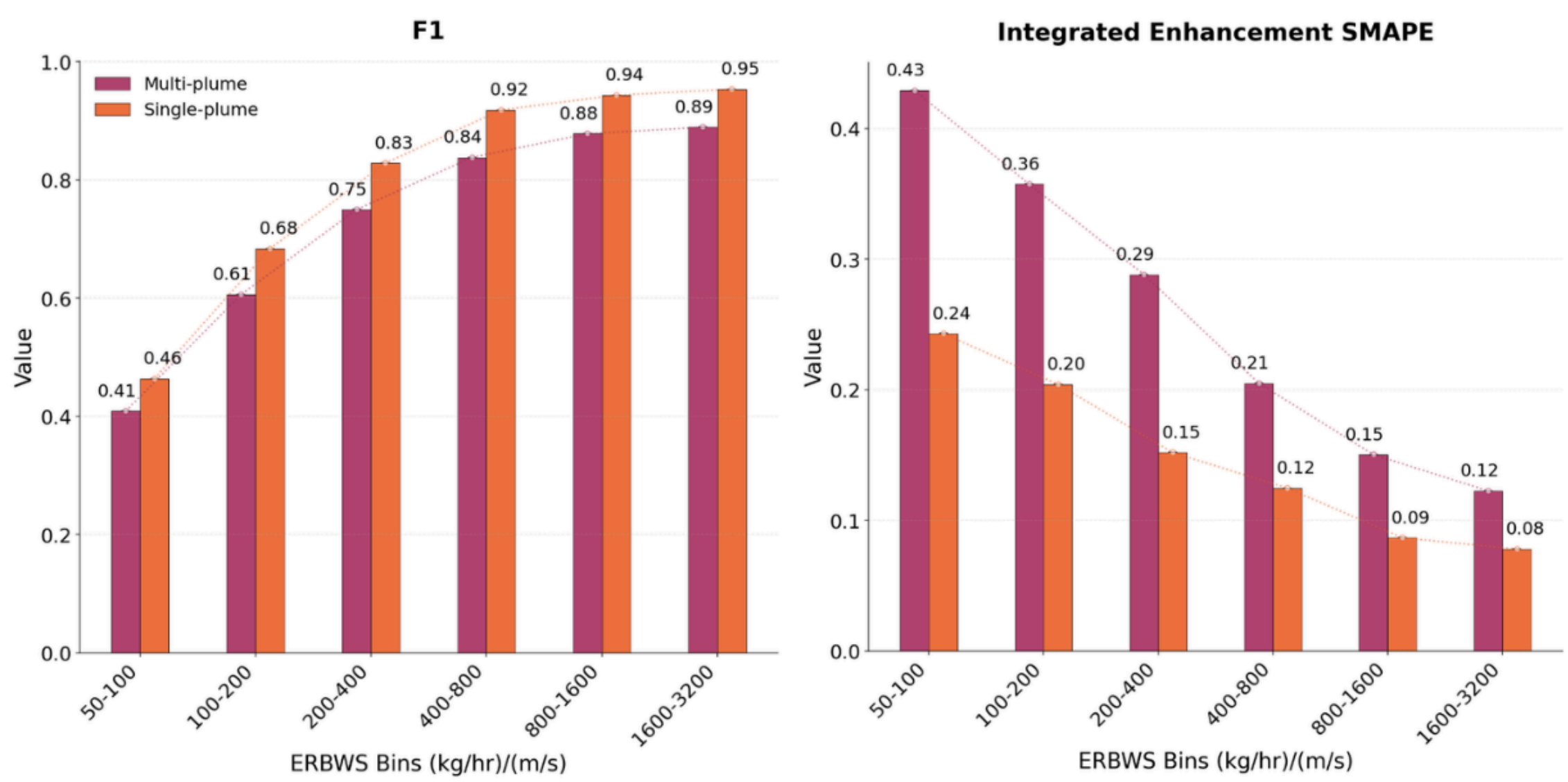

Figure S2: High-fidelity detection and enhancement mass quantification of MAPL-EMIT methane plumes. The figure presents the F1 score (left) and integrated enhancement SMAPE (right) stratified by ERBWS buckets. Integrated enhancement SMAPE across the synthetic test split demonstrates the model's capacity to accurately estimate the total plume mass essential for emission rate quantification.

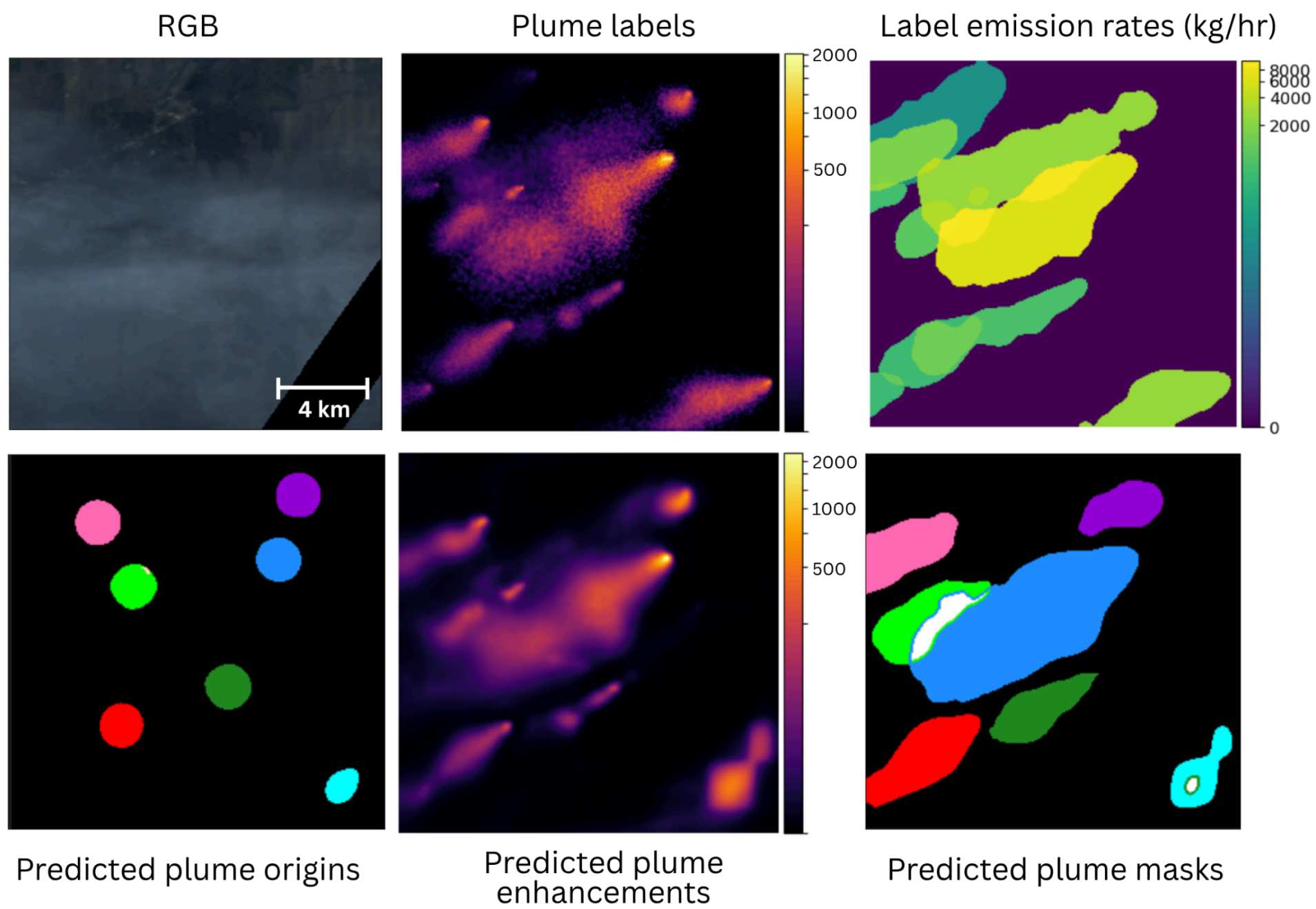

Figure S3: MAPL-EMIT performance on synthetic data. This example demonstrates the model's ability to disentangle overlapping plumes and accurately identify origins under varying emission rates. Each row displays (from left to right): RGB, ground-truth plume labels with mask outlines, predicted source locations, aggregated model enhancements, and predicted instance masks where overlapping segments are highlighted in white.

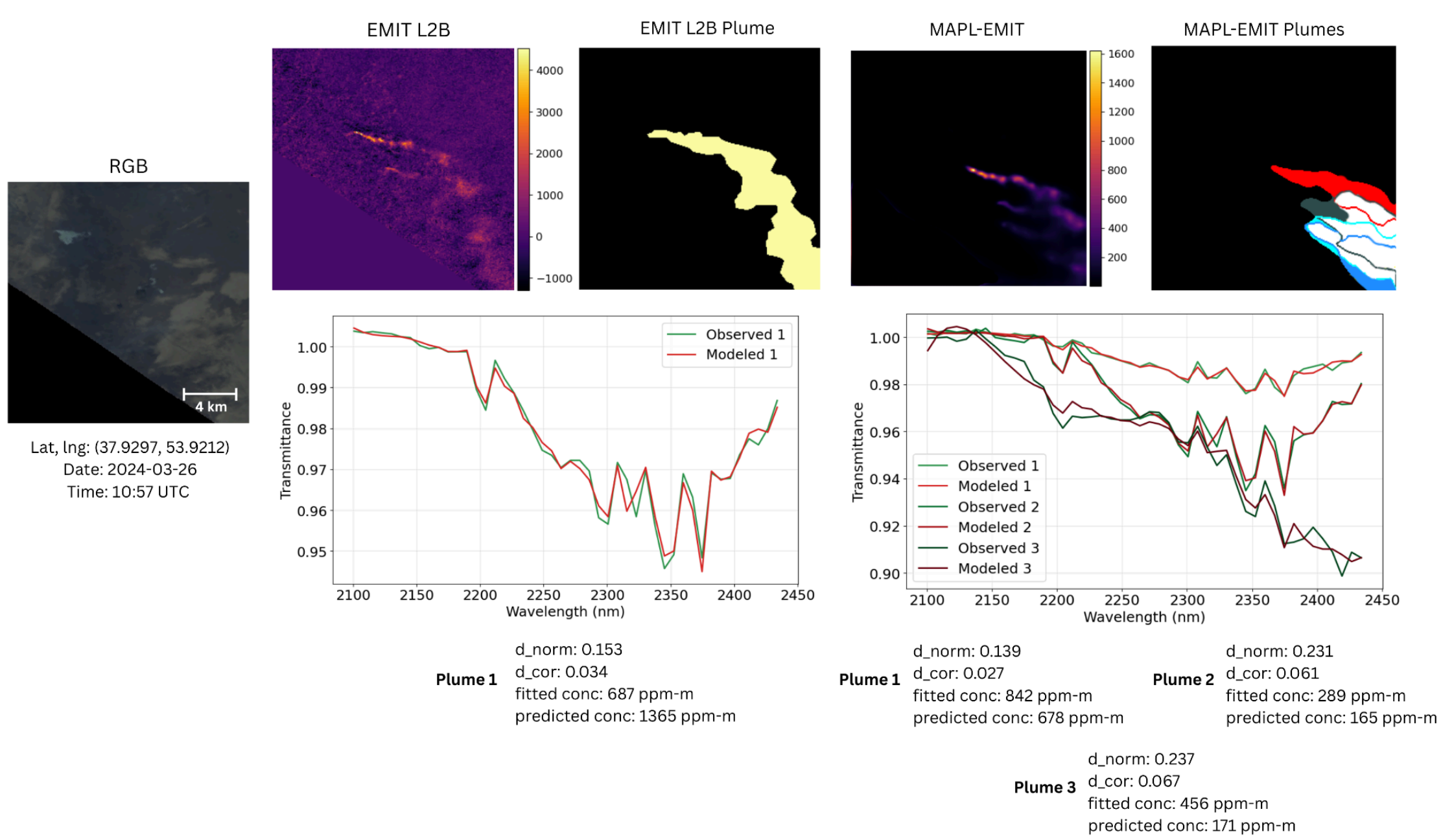

Figure S4: Comparative visualizations of plume detections across MAPL-EMIT predictions and EMIT L2B. From left to right: the RGB image, EMIT L2B enhancements, EMIT L2B plumes, MAPL-EMIT predicted enhancements, MAPL-EMIT instance masks, EMIT L2B spectral fit and MAPL-EMIT spectral

fits. This example illustrates the case where both MAPL-EMIT and the operational EMIT L2B identify plumes but MAPL-EMIT picks up additional plumes.

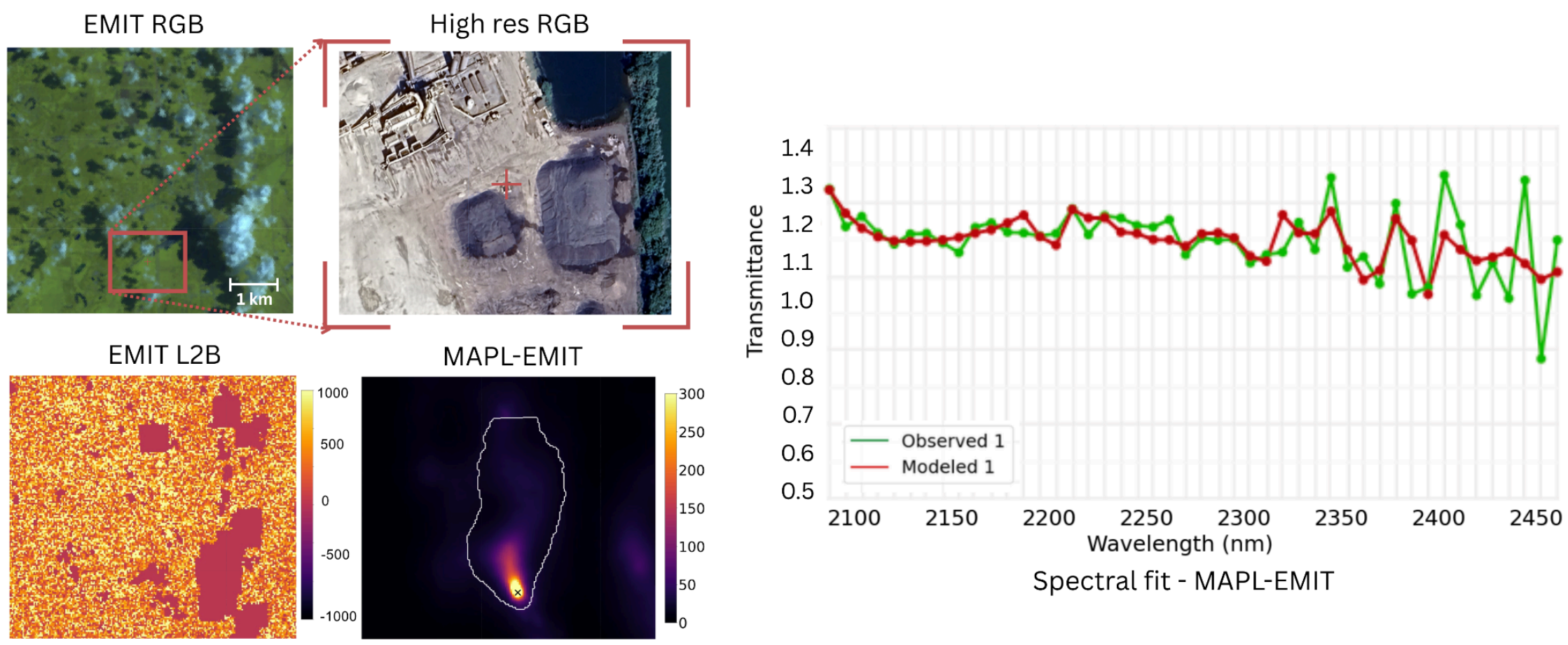

Figure S5: MAPL-EMIT identifies significant methane plumes that are occasionally missed by manual NASA L2B analysis. This example from a concrete/asphalt facility (29.153079, -95.436677) highlights a potential high-intensity plume detected by MAPL-EMIT that was omitted from the NASA L2B catalog, likely due to human omission or the presence of partially obstructing clouds and low-contrast enhancements in the matched-filter output.

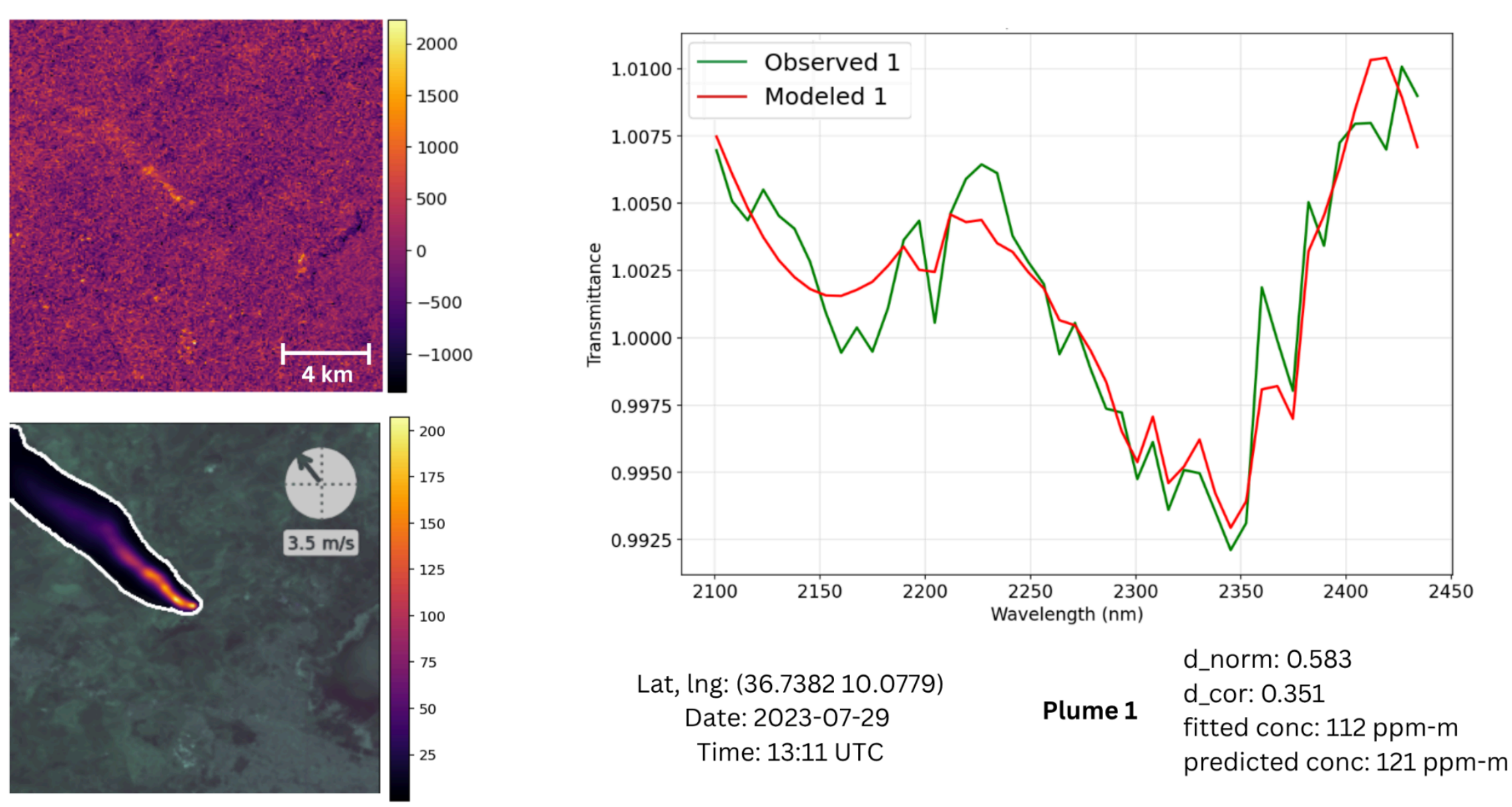

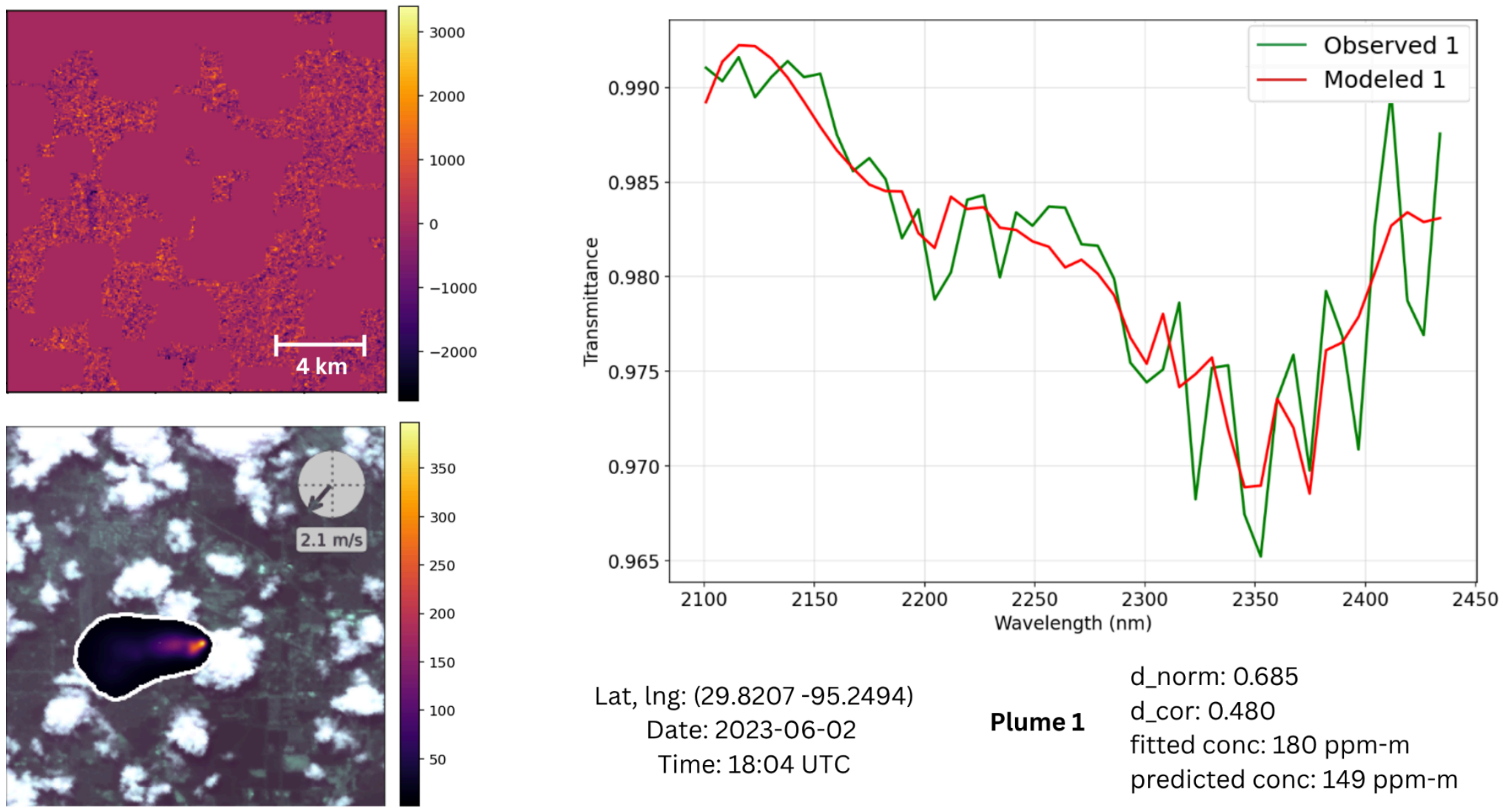

Figure S6: MAPL-EMIT achieves robust methane plume detection across complex and heterogeneous landfill environments globally. The panels display NASA L2B matched-filter enhancements, and the corresponding MAPL-EMIT predicted plume masks and enhancements overlaid on the EMIT RGB imagery along with a small compass on the top right showing the ERA-5 10m wind direction and the magnitude for some of the top-emitting landfills, demonstrating robust detection even under partial cloud cover.

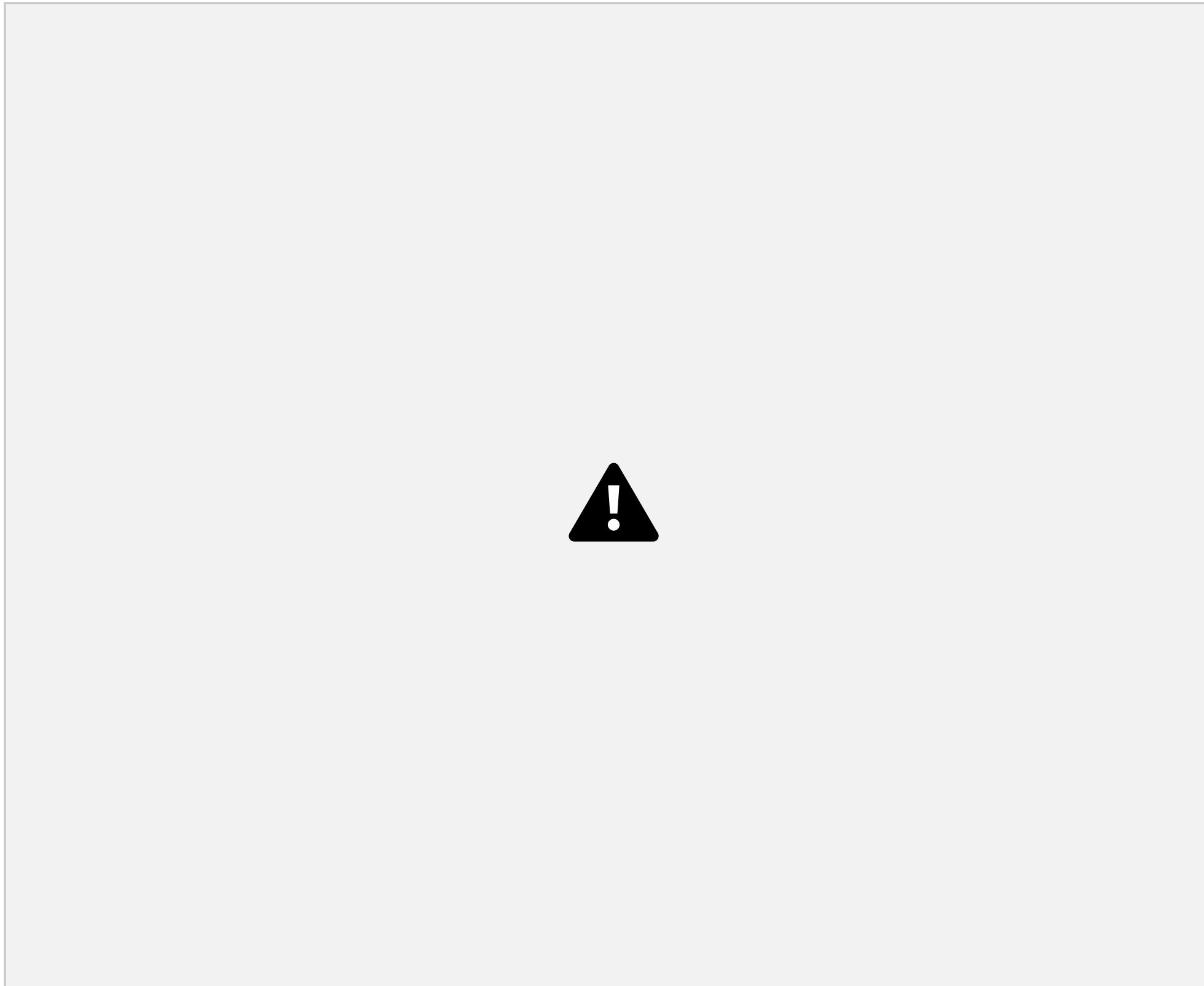

Figure S7: Temporal monitoring at the Amman landfill reveals persistent emission events and varying plume dispersion patterns. This time-series of detections from August 2022 to July 2025 shows active methane plumes (colored) overlaid on a cumulative history of prior detections (grey shadows), illustrating the variability in plume morphology driven by fluctuating local winds from ERA-5 10m (bottom left of each image) over a known persistent source, also indicated in the image on the top right.

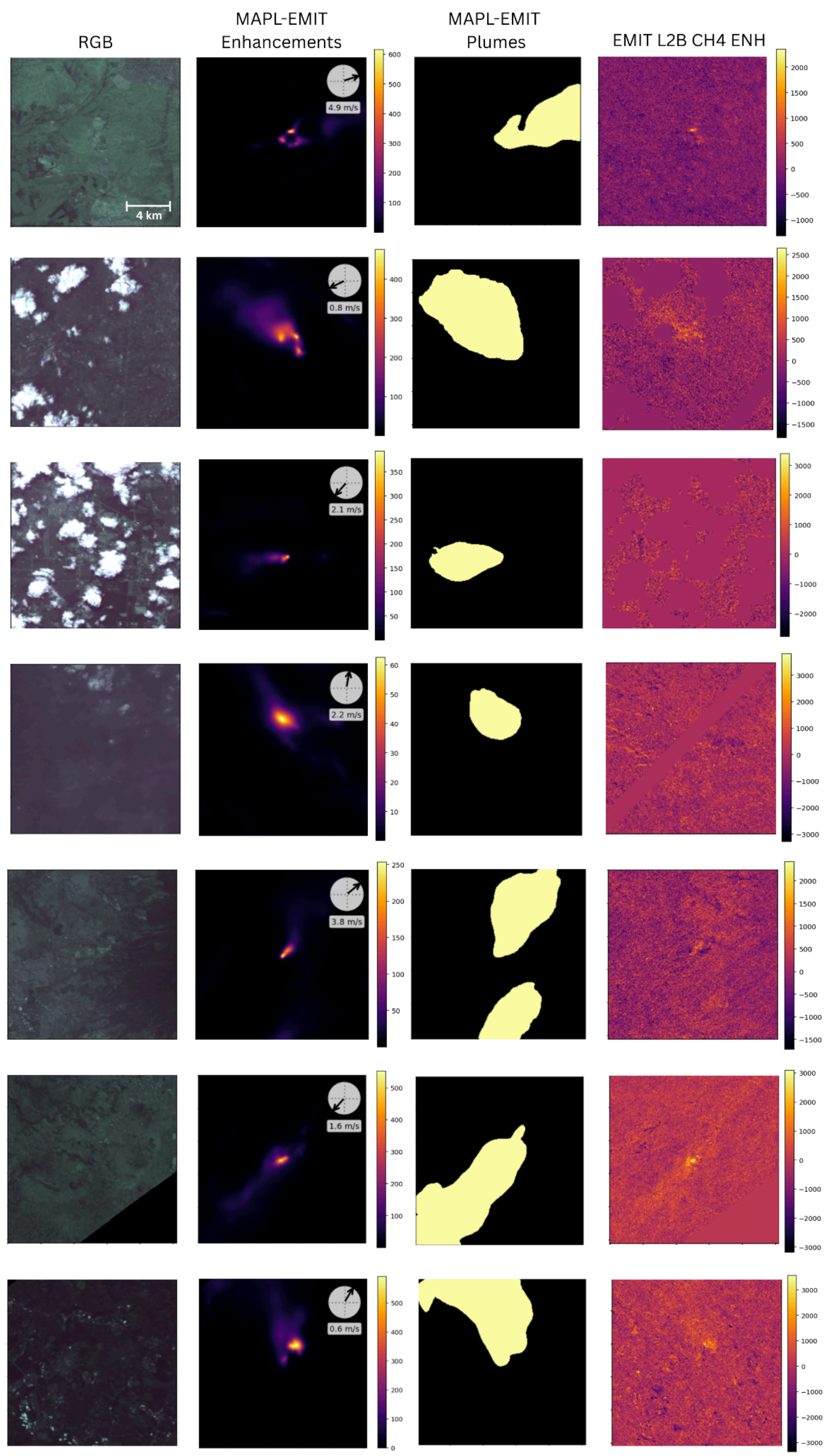

Figure S8: Landfill sites where methane plumes are hard to spot in EMIT L2B enhancements but are clearly identified by MAPL-EMIT. Each row corresponds to one location. The MAPL-EMIT enhancement contains the wind direction on the top right as well. For samples 1, 6, and 7, the plume extent and direction are difficult to discern from L2B enhancements; in samples 2, 3, and 4, identification is hindered

by masked regions in the standard L2B data; and in sample 5, the plume is not visible in the L2B enhancement due to a low SNR.

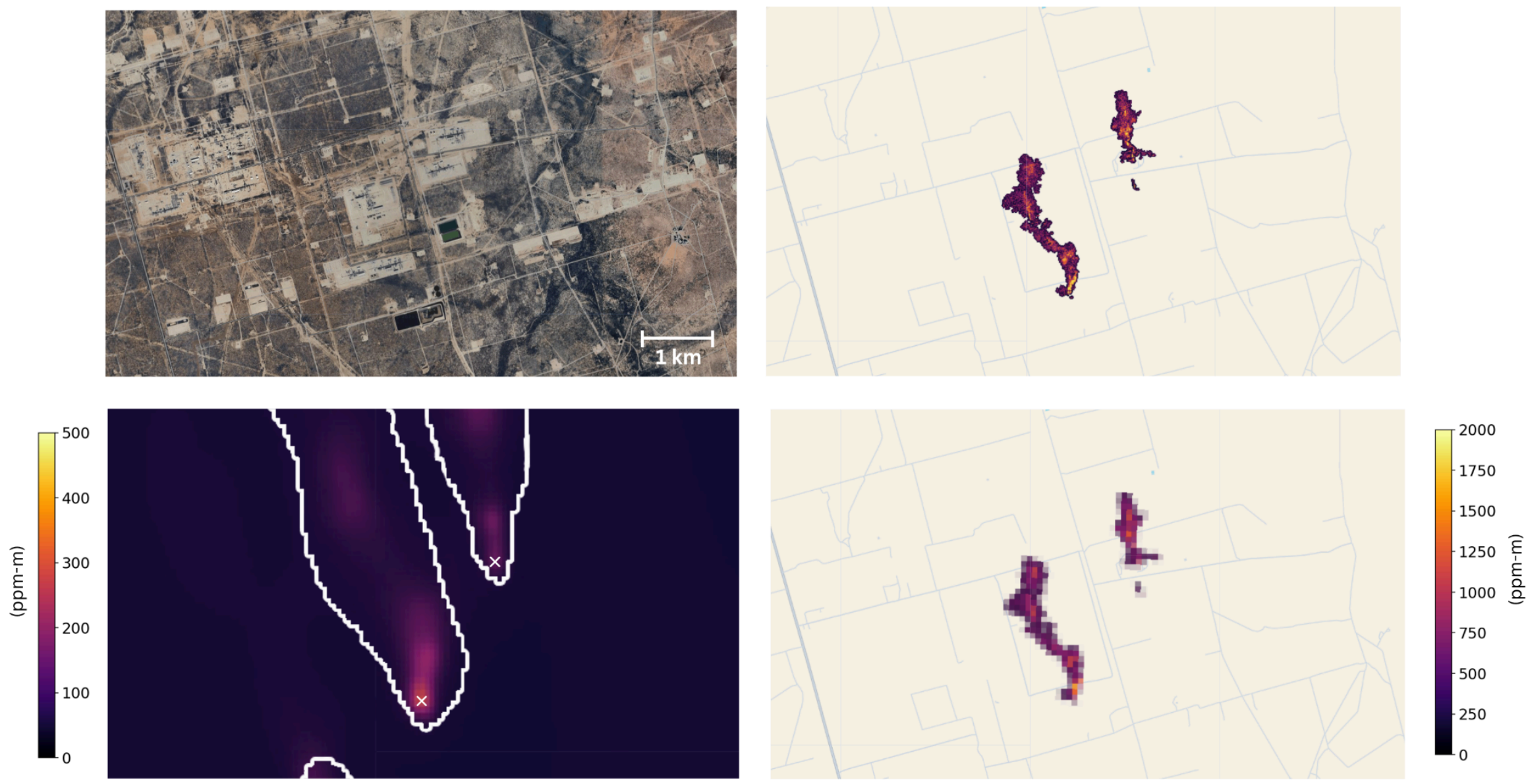

Figure S9: Coincident airborne observations from the Global Airborne Observatory (GAO) validate the accuracy of EMIT-based detections. The panels show high res RGB imagery, GAO full resolution plumes, MAPL-EMIT enhancements with plume outlines and GAO plumes resampled to 60m spatial resolution to match EMIT. Qualitative verification of EMIT-based detections against coincident GAO airborne data at multiple locations shows the model's ability to accurately reconstruct plume morphology and capture enhancements confirmed by the higher-SNR aerial data.

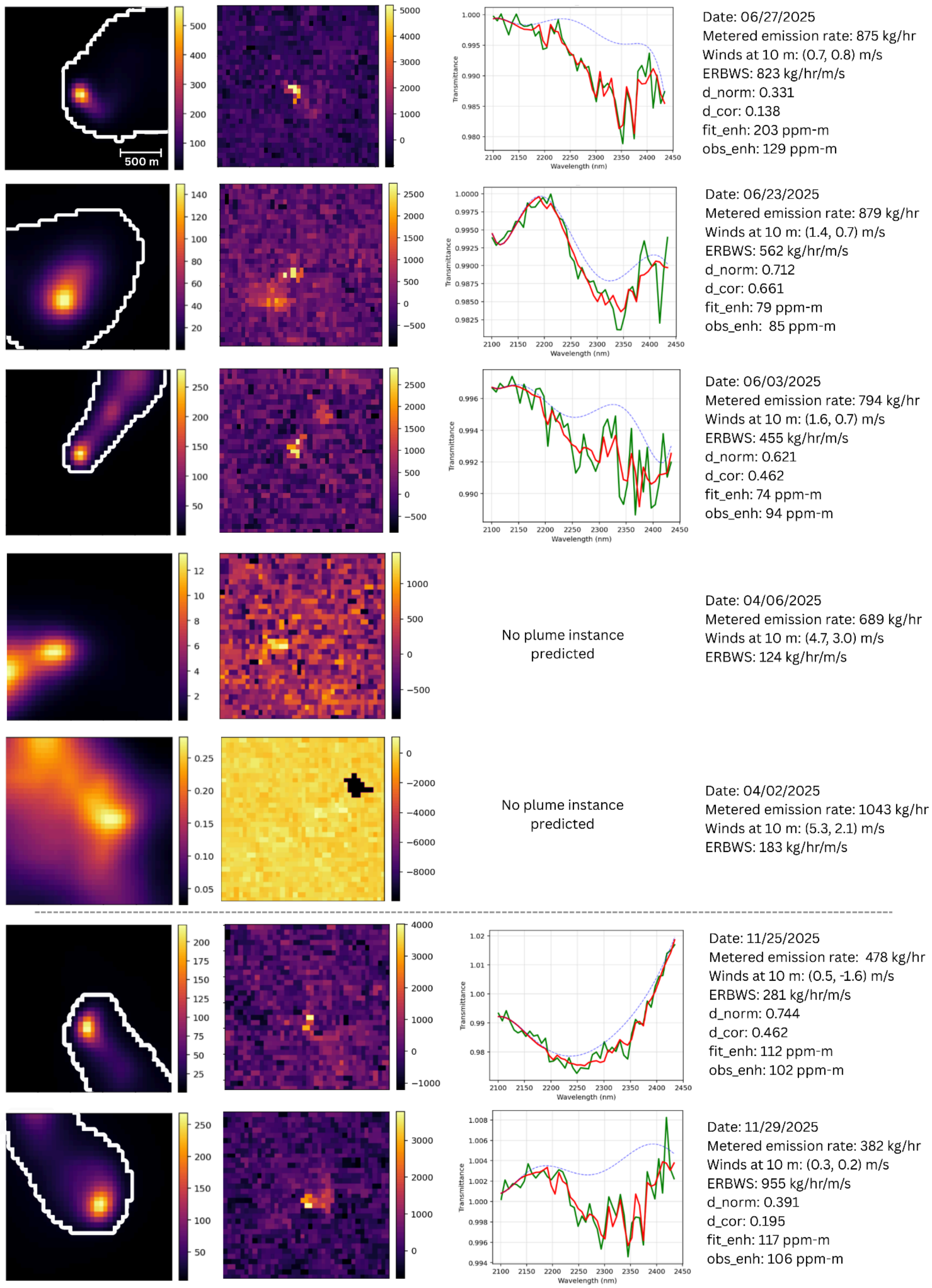

Figure S10: Controlled release experiments validate MAPL-EMIT's detection sensitivity and physical consistency. The figure compares MAPL-EMIT enhancements and plume masks against NASA L2B enhancements for seven controlled methane releases, showing successful detection of medium-intensity plumes with physical validity confirmed by independent spectral fit metrics.

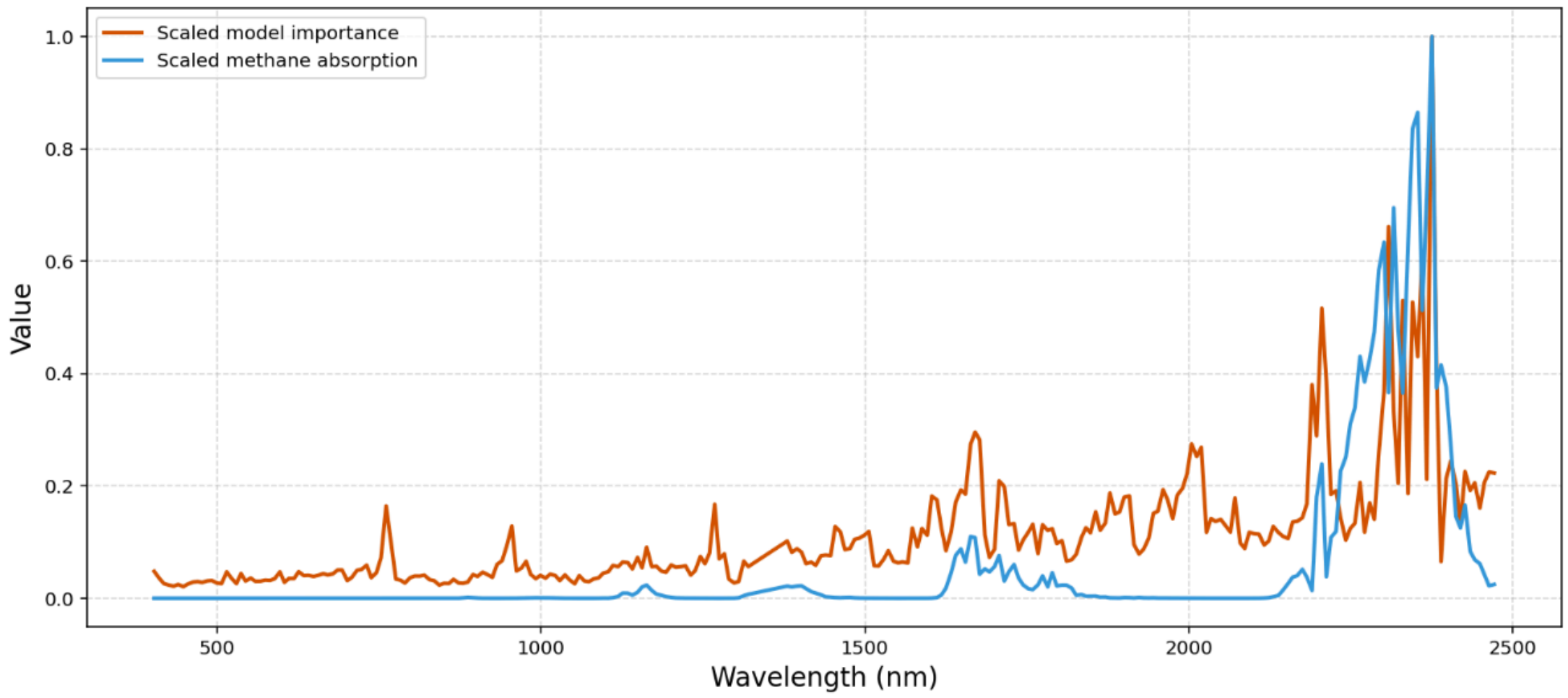

Figure S11: The deep learning model prioritizes physically relevant spectral bands associated with methane absorption manifolds. A comparison between learned patch embedding weights and the methane absorption curve shows that the model's attention peaks align with the three primary methane absorption features in the SWIR, confirming it effectively leverages relevant physical signals.

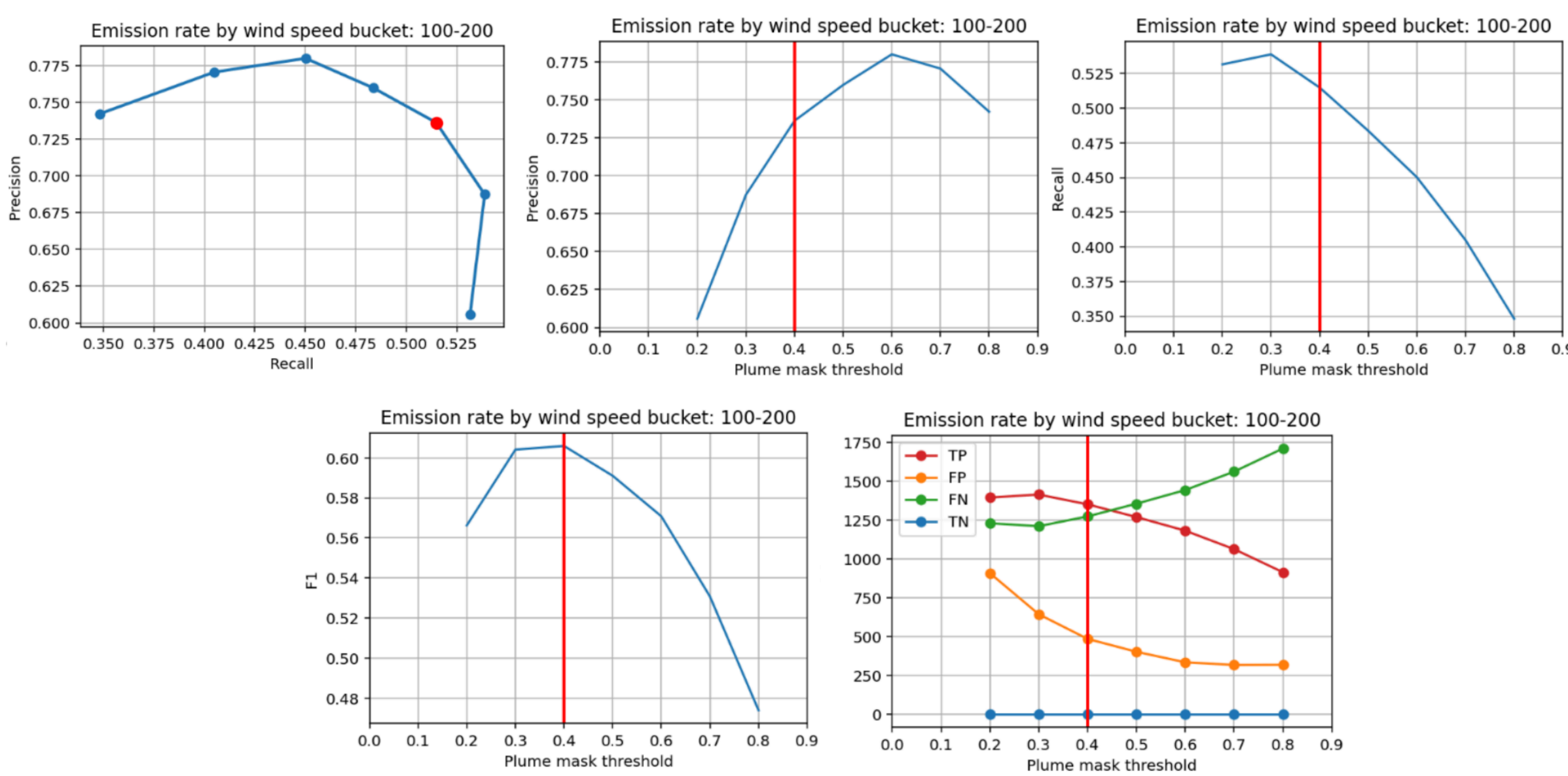

Figure S12: Precision and recall trade-offs can be optimized by adjusting the model's plume probability threshold. The top row displays the Precision-Recall (PR) curve and the relationship between classification outcomes across varying thresholds, while the bottom row shows F1, precision, and recall for the 100-200 ERBWS bucket, justifying the selection of an operational threshold.

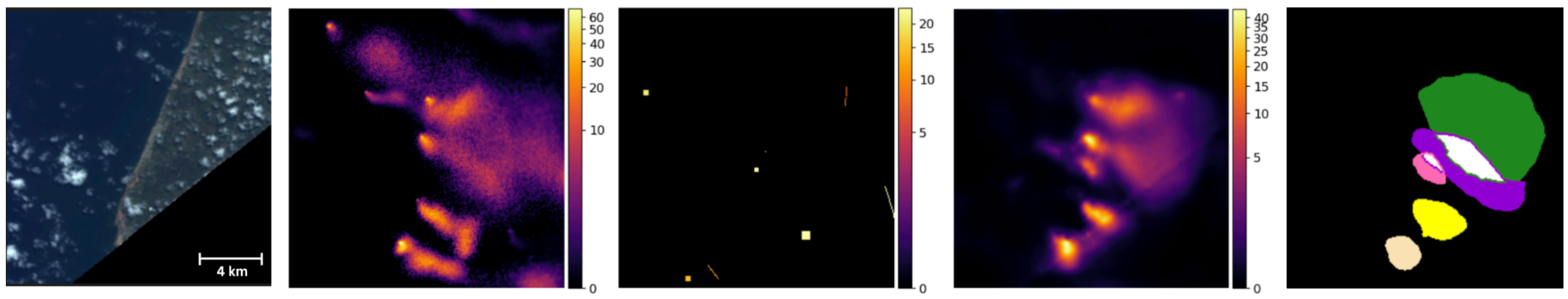

Figure S13: MAPL-EMIT effectively discriminates genuine methane plumes from non-physical surface artifacts. The panels display (from left to right): RGB context imagery, ground-truth plume labels, simulated surface artifacts designed to mimic methane's spectral signature but lacking realistic plume morphology, model-predicted methane enhancements, and predicted plume masks. The model successfully suppresses these non-physical spectral anomalies, accurately identifying genuine plumes while ignoring the simulated surface noise.

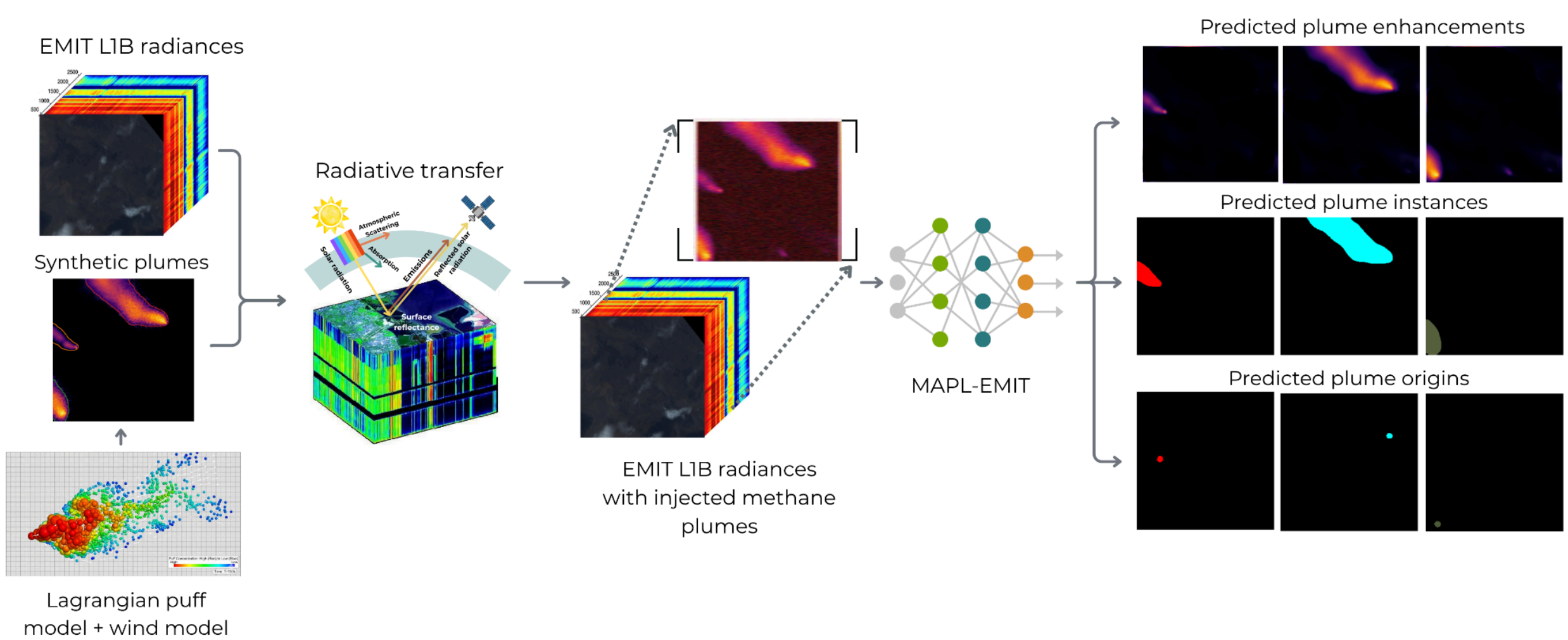

Figure S14: The MAPL-EMIT model utilizes a comprehensive synthetic-to-real training pipeline to overcome data scarcity. This methodological overview illustrates the integration of EMIT L1B radiances with synthetic plumes generated via a Lagrangian puff model and physics-based radiative transfer, forming the basis for training the joint prediction heads.

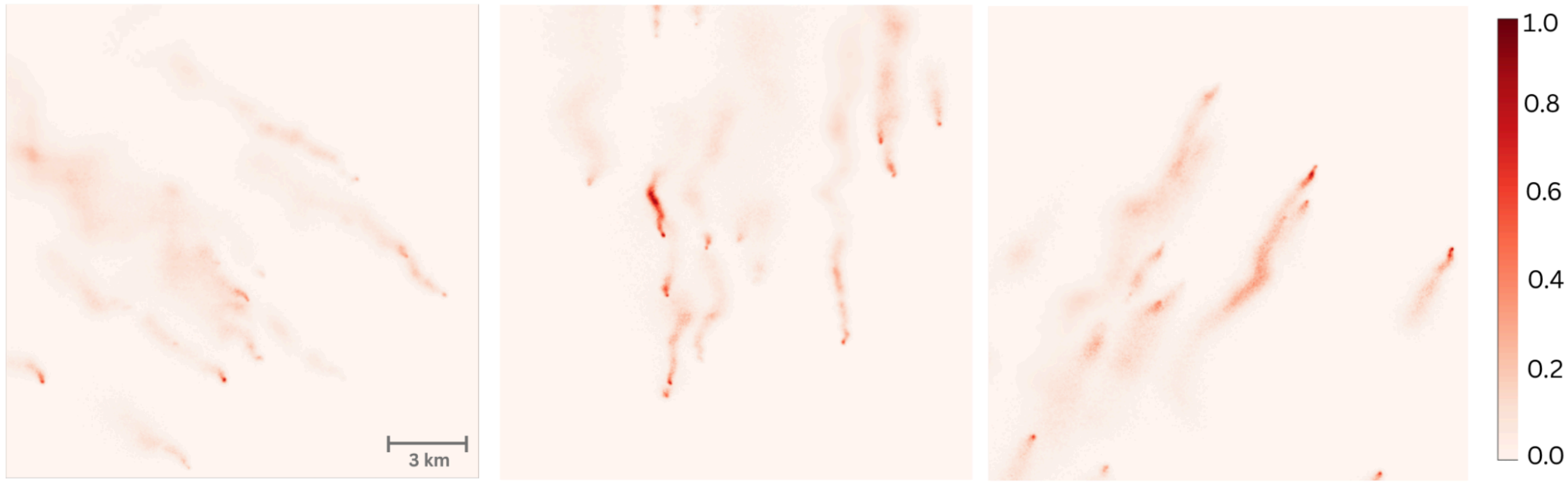

Figure S15: Lagrangian puff simulations capture the complex turbulence and intermittency of real-world methane release events. Examples of synthetic 60m-resolution plume tiles show the range of morphologies and intensities used during training, providing the high-fidelity ground truth necessary for the model to learn spatial and spectral gas features.

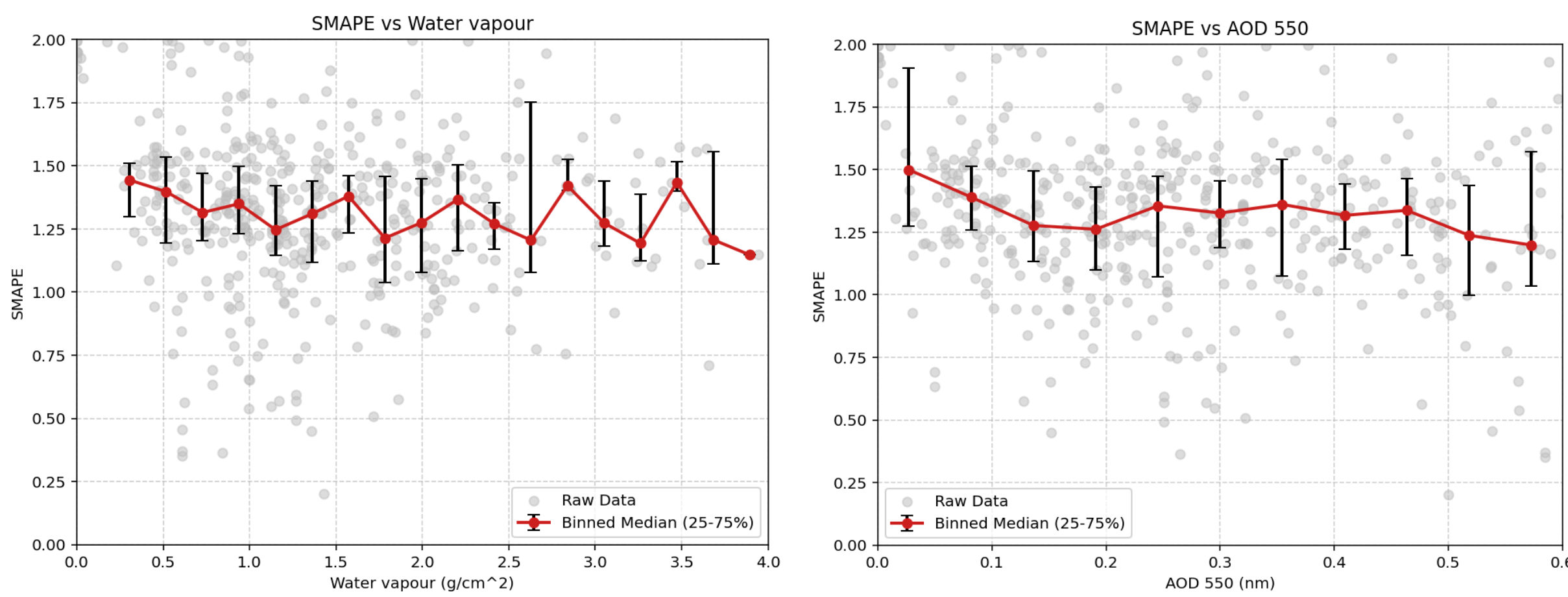

Figure S16: MAPL-EMIT methane retrievals remain robust across a wide range of water vapor and aerosol concentrations. Plots of integrated enhancement SMAPE versus water vapor column density (left) and Aerosol Optical Depth (right) on the NASA plumes dataset show no significant correlation between retrieval error and these atmospheric variables.

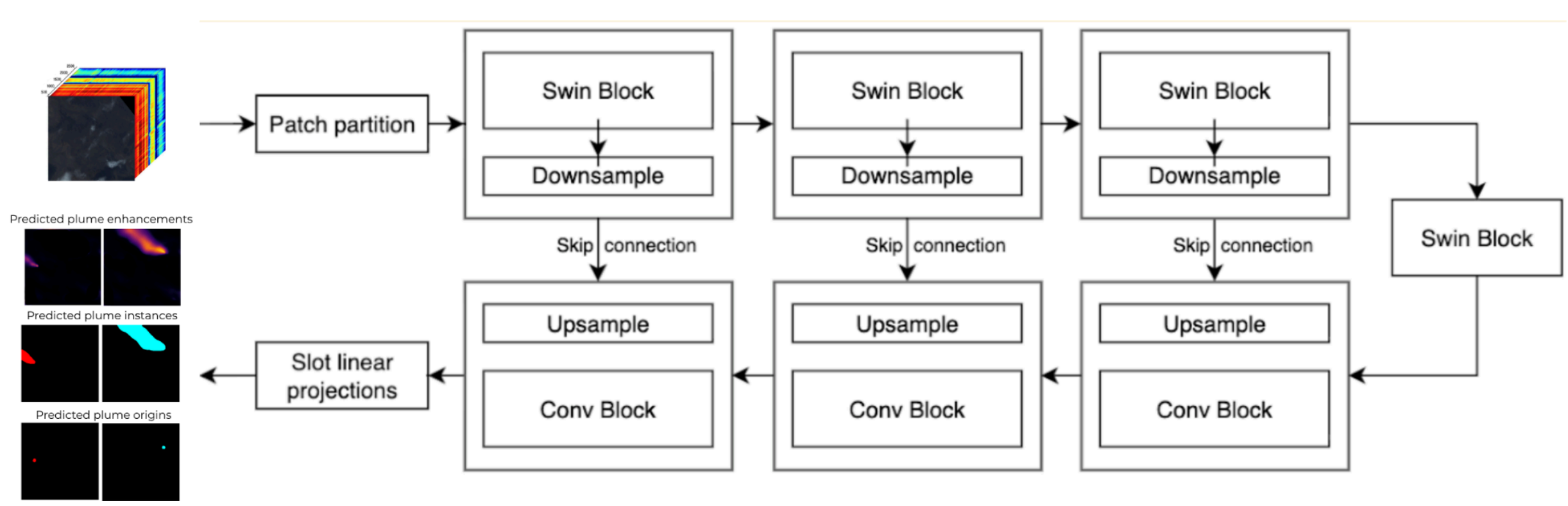

Figure S17: A U-Net-like architecture with a Swin Transformer encoder enables multi-scale feature extraction for methane retrieval. The diagram details the model structure, featuring a Swin-v2-S encoder for feature extraction from hyperspectral inputs and a convolutional decoder with skip connections that output parallel predictions for enhancements, masks, and origins.

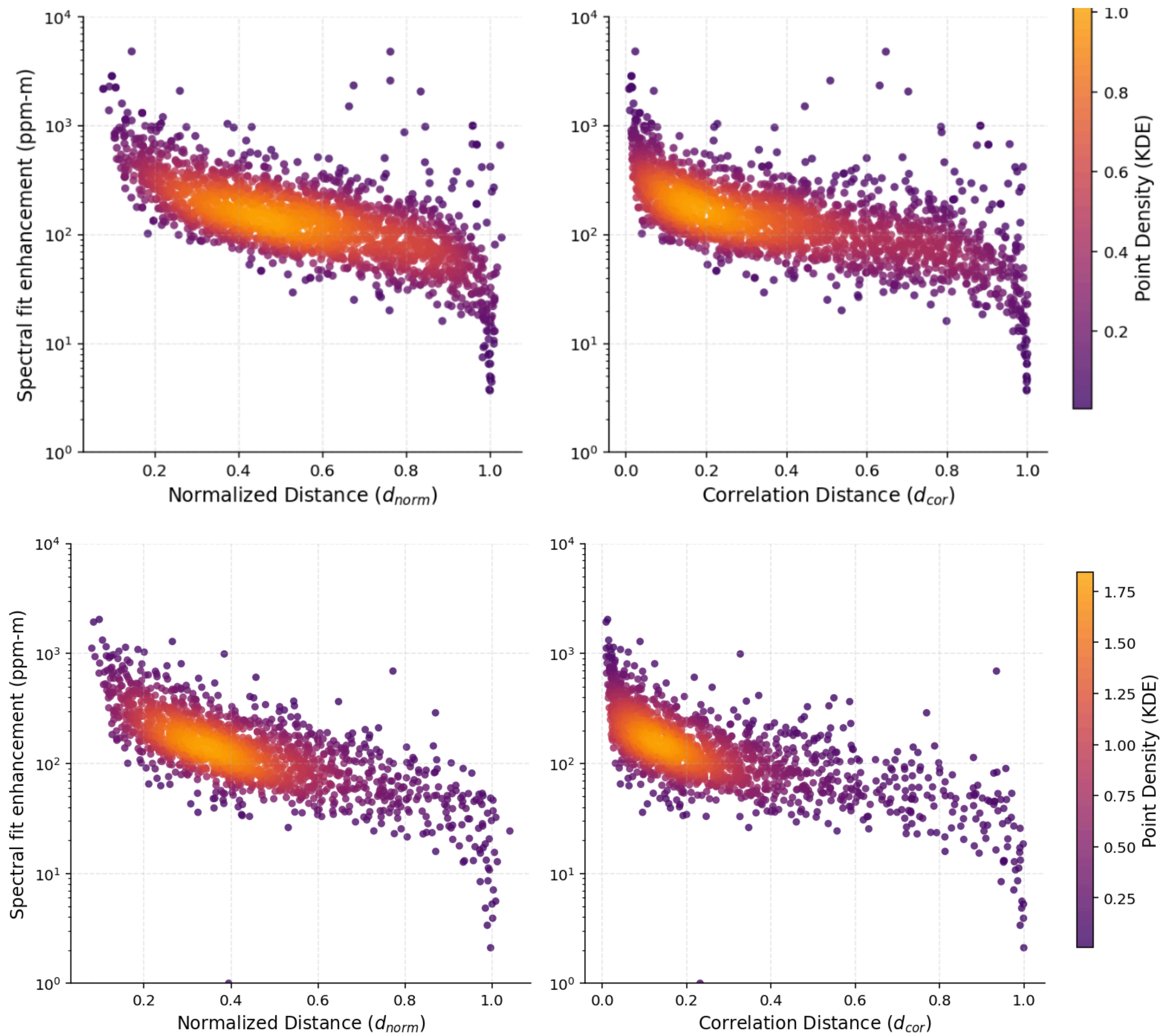

Figure S18: Spectral fit metrics provide independent physical verification of real-world methane plume detections. Distributions of normalized distance (d_norm) and correlation distance (d_cor) for MAPL-EMIT (top) and NASA L2B (bottom) across identical granules show that the model's detections consistently align with expected methane absorption manifolds.

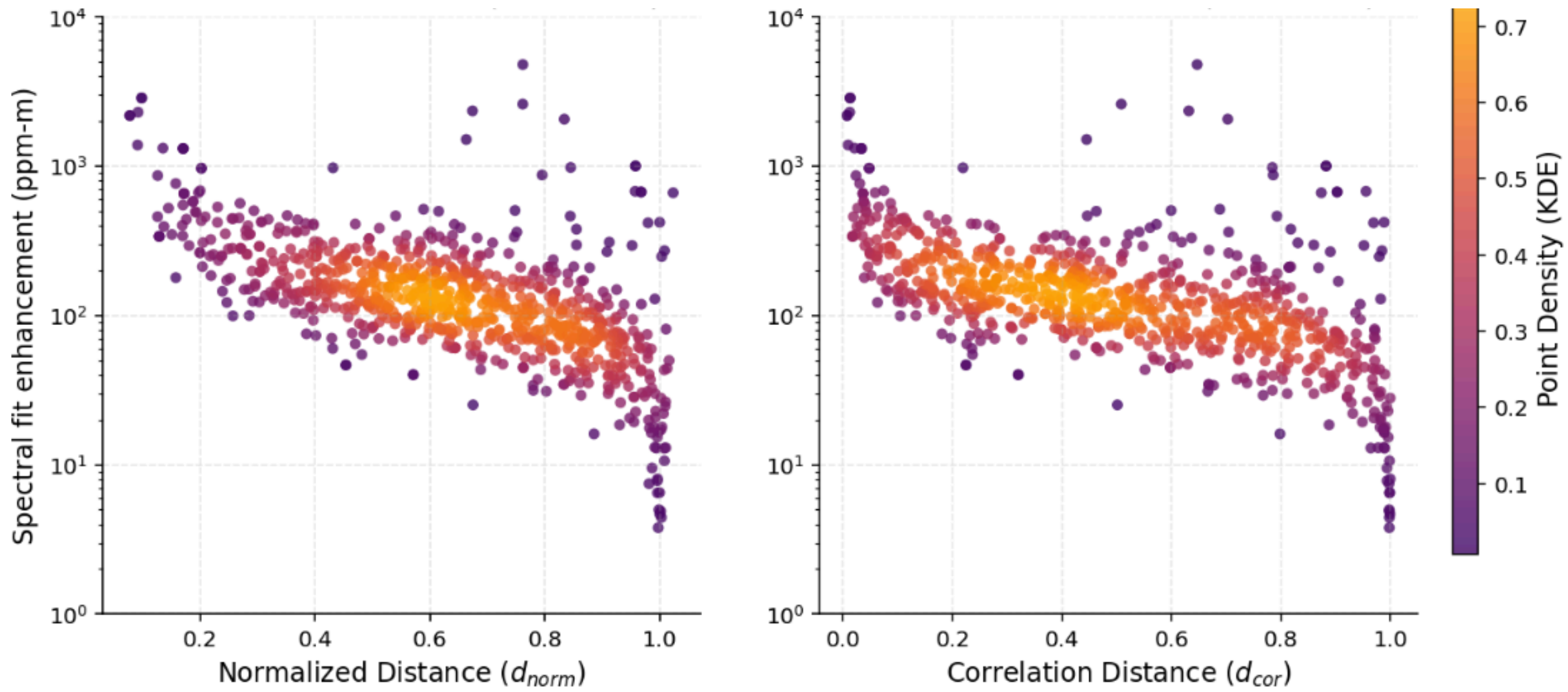

Figure S19: MAPL-EMIT identifies hundreds of plausible methane plumes missed by established manual catalogs. Spectral fit analysis of plumes detected by MAPL-EMIT but absent from the NASA L2B catalog indicates that approximately 67% exhibit spectral signatures consistent with genuine methane emissions.

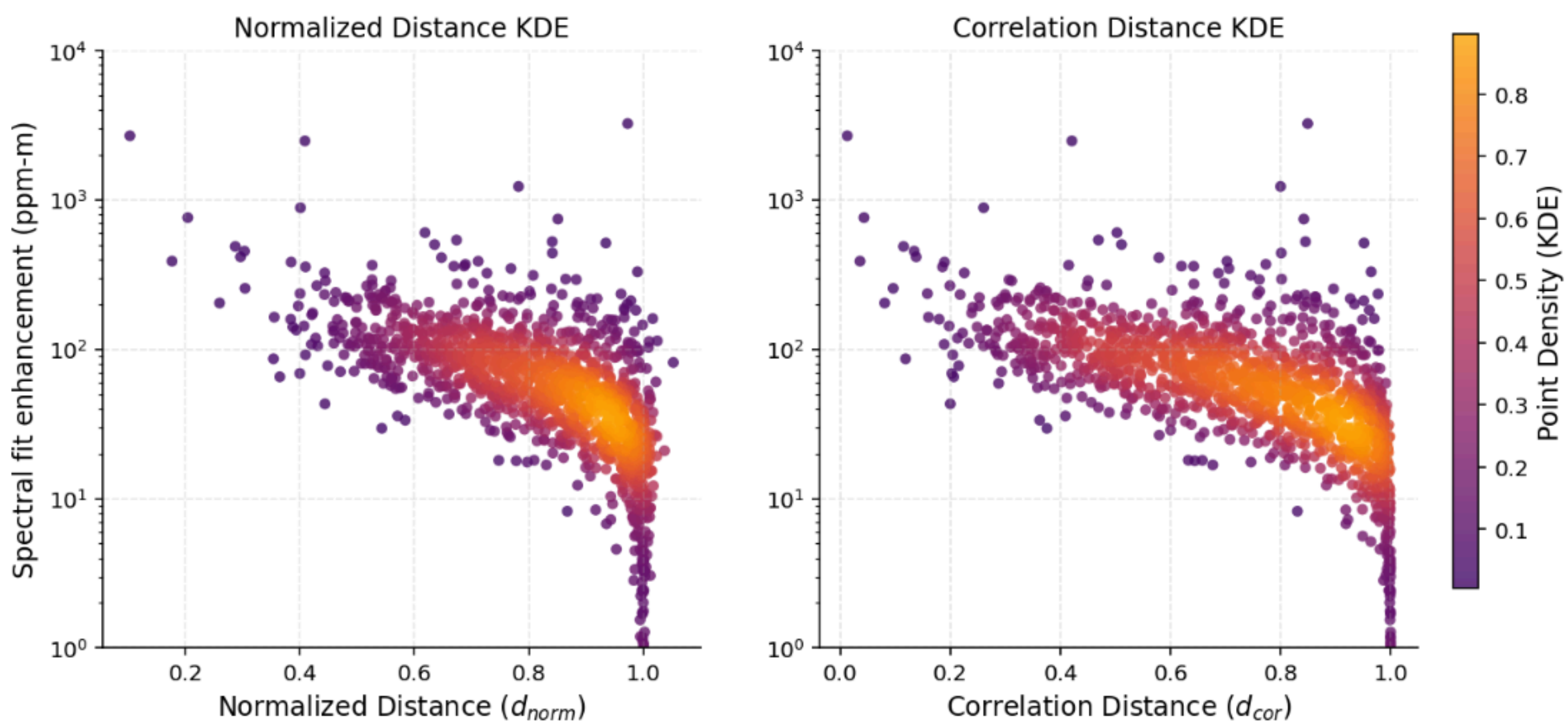

Figure S20: Evaluation on granules with no identified plumes from NASA provides a conservative estimate of the model's false-positive rate. Analysis of 1,513 detections in 20,000 granules expected to be plume-free shows that 355 are potentially real emissions based on spectral fit metrics, with the remainder defining the limit of retrieval artifacts.

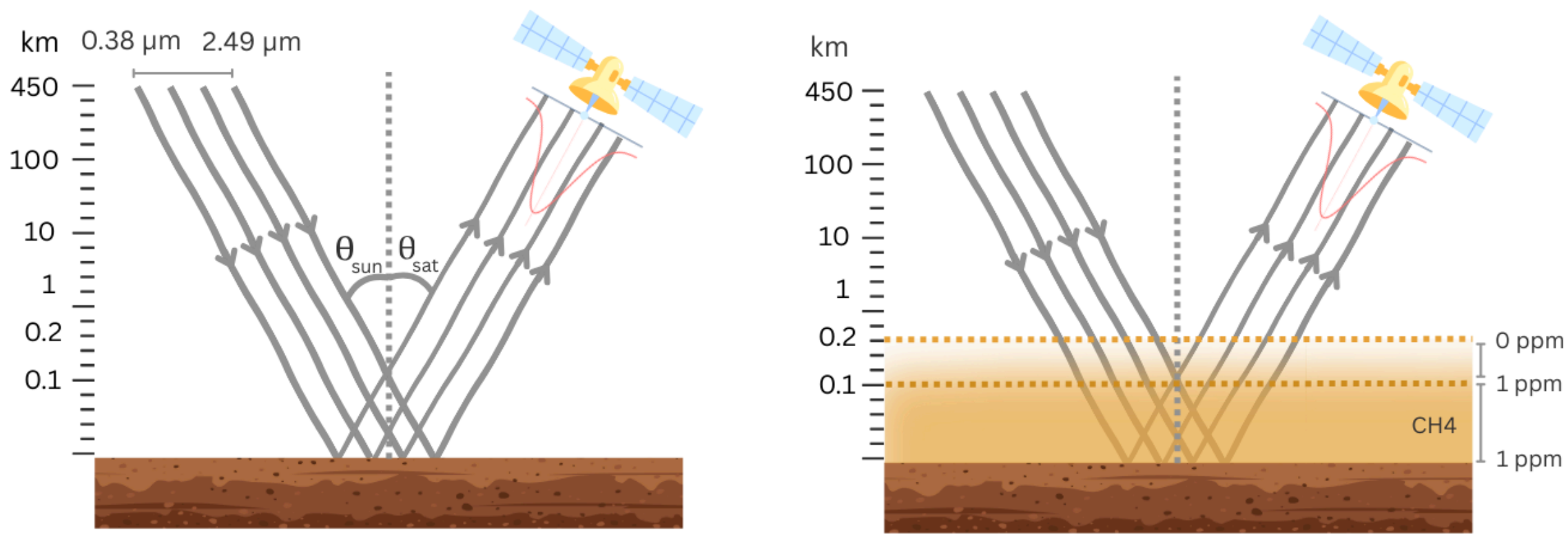

Figure S21: Radiative transfer modeling accounts for column-integrated absorption along the solar and satellite photon paths. The illustration depicts the simplified Beer-Lambert absorption model used to calculate transmittances for background and methane-enhanced atmospheres, accounting for total path length as a function of viewing geometry.

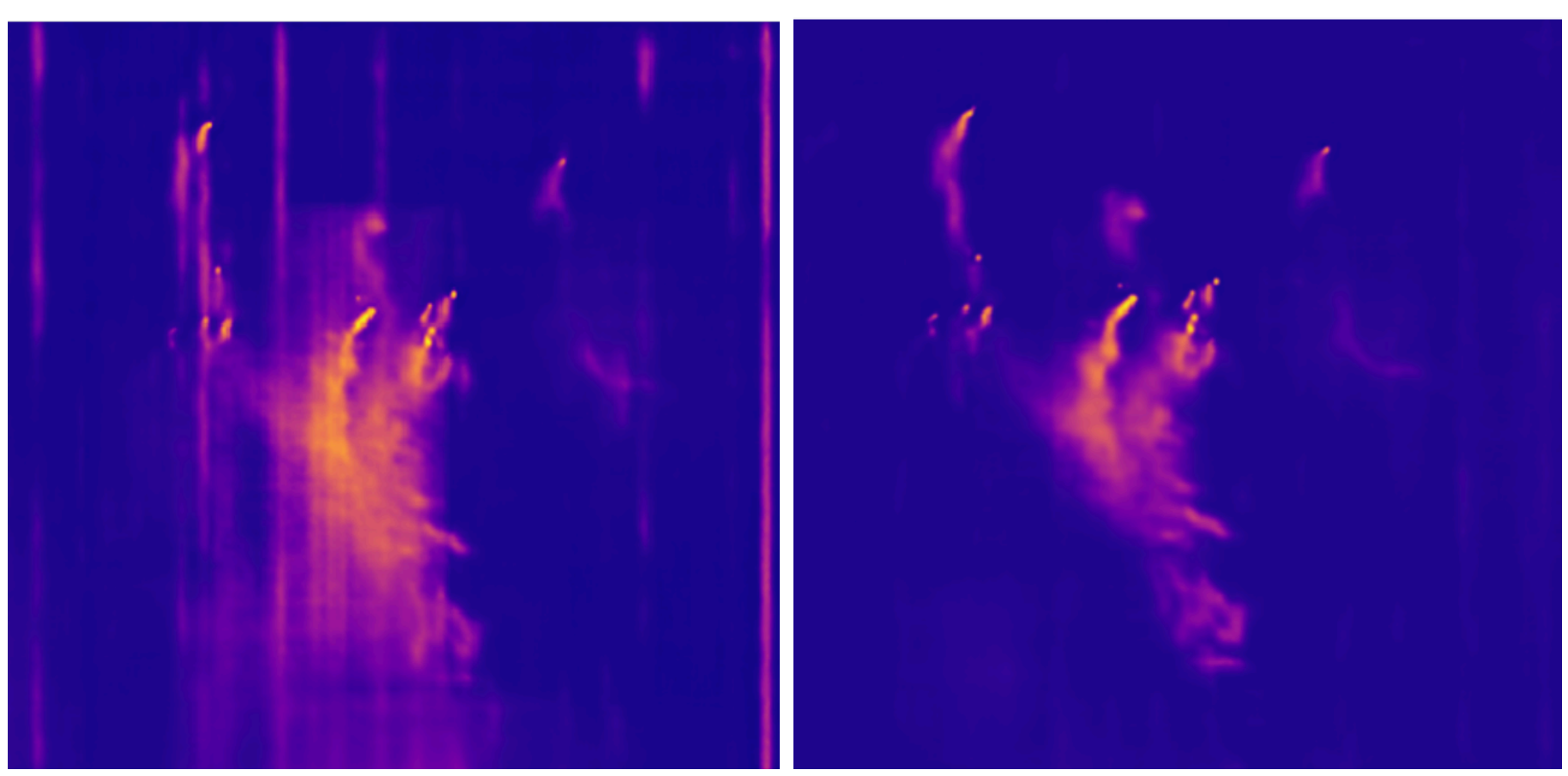

Figure S22: Cross-track radiometric correction effectively mitigates detector-level striping artifacts in EMIT granules. A comparison of model outputs with and without cross-track normalization demonstrates that correcting for systematic illumination differences across the sensor swath prevents the hallucination of features tied to detector geometry.

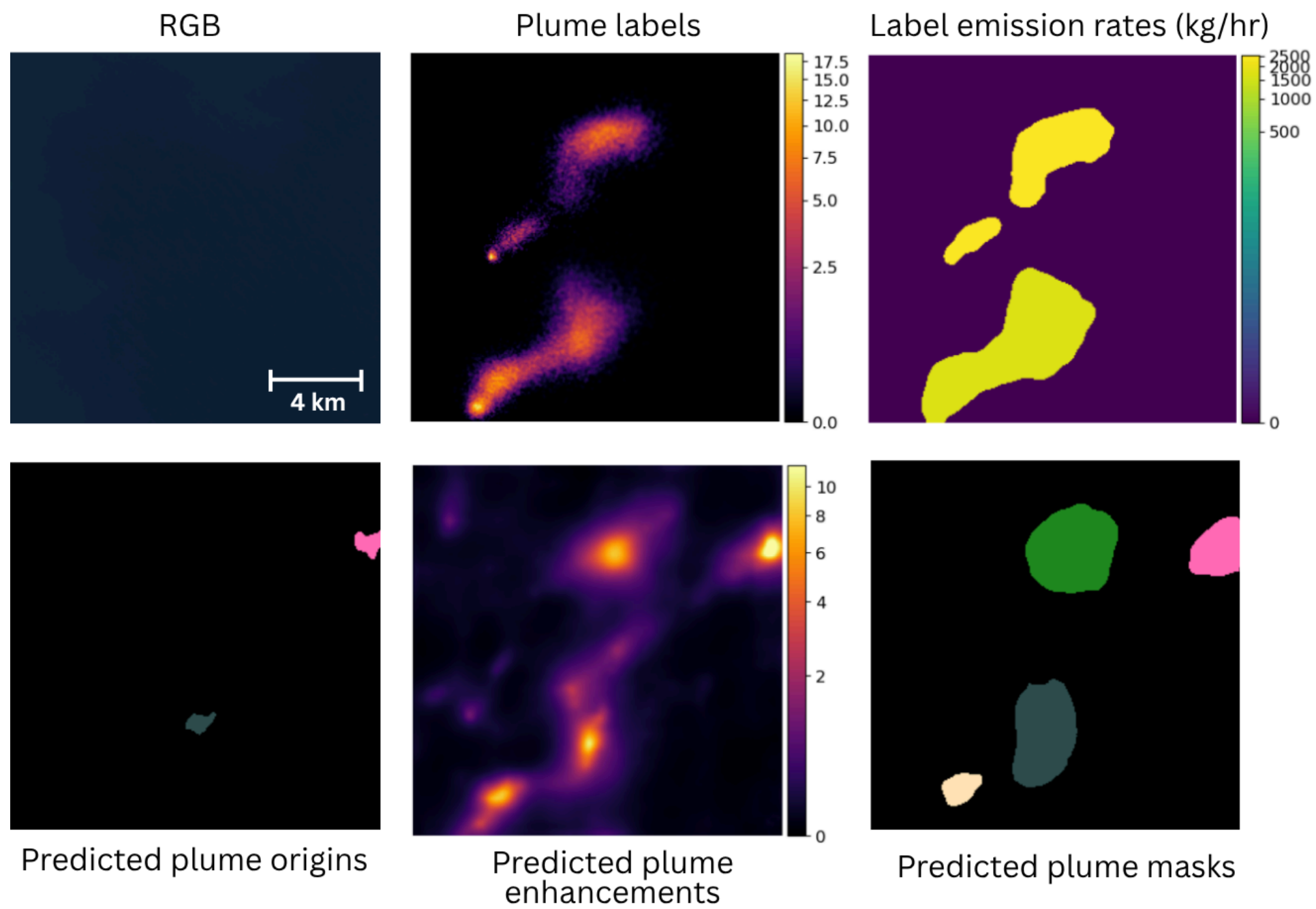

Figure S23: Identification of characteristic failure modes guides future improvements to the deep learning framework. Representative examples of retrieval errors include fragmented masks for highly intermittent plumes over low-reflectance terrain and challenges in perfectly delineating plumes in scenarios with extreme spatial overlap.

Tables

Table S1: Hyperparameters used for plume simulation. This table lists the key parameters and their corresponding values utilized in the generation of the synthetic plume dataset.

**Model**	**H-param**	**Description**	**Value / Uniformly sampled from**
Plume model	Num plumes	The number of plumes in a tile	(1, 10)
	Simulation duration	The duration of the total simulation	3000 seconds
	Plume duration	The duration each plume is emitting for. Note that every plume ends emitting at the end of the simulation itself, so the start of the plume emission is set to Simulation duration - Plume duration	Uniformly pick a bucket and then uniformly sample from the bucket. Buckets:<br>(250, 550),<br>(550, 1200),<br>(1200, 2400),<br>(2400, 3000)
	Simulation region	The size of the simulation region. This region is then cropped (random crop during training, center crop during eval) to 256x256 pixels which is the size of each EMIT tile.	348x348 pixels at 60m resolution. Total region size = 23,040
	Center relative diff scale	Each puff moves with x' = x + (u + rand * center_relative_diff_scale) *dt, this adds noise into the wind field for each puff at every timestep	(10, 20)
	Puff initial radius	The initial radius of the puff spawned in m	(3, 20)
	Puff spread rate	The puff's radius increases by pow(puff_spread_rate, dt) every timestep	(1.002, 1.003)
Wind model	Mean wind speed	Initial mean wind speed in m/s across the wind field	(0, 10)
	Velocity clip	Maximum wind speed across the wind field across time	30
	Diffusivity range	Diffusivity constant	(30, 50)
	Puff release rate	Number of puffs released for each plume from the origin	Mean = 20, std = 4

Table S2: Impact of output scaling on retrieval performance. Analysis of scaling methods conducted on the synthetic multi-plume validation split, focusing on the 100–200 ERBWS stratification bucket. Note that the ablation was performed on stage 1 of training (500k steps with 128 batch size).

**Output scaling**	**F1**	**Precision**	**Recall**
**Sqrt (Baseline)**	**0.609**	**0.763**	0.507
Linear	0.569	0.712	0.474
Log1p	0.605	0.746	**0.508**

Table S3: Impact of removing aggregate losses on retrieval performance. Analysis was conducted on the synthetic multi-plume validation split, focusing on the 100–200 ERBWS stratification bucket. Note that the ablation was performed on stage 1 of training (500k steps with 128 batch size).

**Experiment**	**F1**	**Precision**	**Recall**
**Baseline**	**0.609**	**0.763**	**0.507**
No aggregate regression loss	0.589	0.757	0.482
No aggregate segmentation losses	0.572	0.742	0.465
No aggregate losses	0.555	0.744	0.442

Table S4: Impact of plume upweight (enhancement, semantic) on retrieval performance. Analysis was conducted on the synthetic multi-plume validation split, focusing on the 100–200 ERBWS stratification bucket. Note that the ablation was performed on stage 1 of training (500k steps with 128 batch size).

**Experiment**	**F1**	**Precision**	**Recall**
**30x, 1x (Baseline)**	**0.609**	**0.763**	0.507
1x, 1x	0.567	0.735	0.461
15x, 1x	0.599	0.754	0.496
50x, 1x	0.604	0.745	0.507
1x, 2x	0.569	0.692	0.483
15x, 2x	0.600	0.714	**0.518**
30x, 2x	0.584	0.705	0.499
50x, 2x	0.598	0.721	0.510

SI References

1. U.S. Standard Atmosphere, 1976 (No. NOAA-S/T-76-1562). Available at https://ntrs.nasa.gov/api/citations/19770009539/downloads/19770009539.pdf
2. L. L. Gordley, B. T. Marshall, D. Allen Chu, Linepak: Algorithms for modeling spectral transmittance and radiance. Journal of Quantitative Spectroscopy & Radiative Transfer 52, 563–580 (1994)
3. D. R. Thompson, R. O. Green, C. Bradley, P. G. Brodrick, N. Mahowald, E. B. Dor, M. Bennett, M. Bernas, N. Carmon, K. D. Chadwick, R. N. Clark, R. W. Coleman, E. Cox, E. Diaz, M. L. Eastwood, R. Eckert, B. L. Ehlmann, P. Ginoux, M. G. Ageitos, … S. Zandbergen, On-orbit calibration and performance of the EMIT imaging spectrometer. Remote Sensing of Environment 303, 113986 (2024)
4. M. Planck, 9. On the distribution law of energy in the normal spectrum. Available at https://web.pdx.edu/~pmoeck/pdf/planck-paper.pdf. Retrieved 6 April 2026
